# Widespread bone-based fluorescence in chameleons

**DOI:** 10.1038/s41598-017-19070-7

**Published:** 2018-01-15

**Authors:** David Prötzel, Martin Heß, Mark D. Scherz, Martina Schwager, Anouk van’t Padje, Frank Glaw

**Affiliations:** 1 Department of Herpetology, Zoologische Staatssammlung München (ZSM-SNSB), Münchhausenstr. 21, 81247 München, Germany; 2 Department Biologie II, Ludwig-Maximilians-Universität München, Großhaderner Straße 2, 82152 Planegg-Martinsried, Germany; 3 Department of Applied Sciences and Mechatronics, Munich University of Applied Sciences, Lothstr. 34, 80335 München, Germany; 4 Department of Ecological Science, Vrije Universiteit Amsterdam, De Boelelaan 1085, 1081 HV Amsterdam, The Netherlands

## Abstract

Fluorescence is widespread in marine organisms but uncommon in terrestrial tetrapods. We here show that many chameleon species have bony tubercles protruding from the skull that are visible through their scales, and fluoresce under UV light. Tubercles arising from bones of the skull displace all dermal layers other than a thin, transparent layer of epidermis, creating a ‘window’ onto the bone. In the genus *Calumma*, the number of these tubercles is sexually dimorphic in most species, suggesting a signalling role, and also strongly reflects species groups, indicating systematic value of these features. Co-option of the known fluorescent properties of bone has never before been shown, yet it is widespread in the chameleons of Madagascar and some African chameleon genera, particularly in those genera living in forested, humid habitats known to have a higher relative component of ambient UV light. The fluorescence emits with a maximum at around 430 nm in blue colour which contrasts well to the green and brown background reflectance of forest habitats. This discovery opens new avenues in the study of signalling among chameleons and sexual selection factors driving ornamentation.

## Introduction

Fluorescence has been reported from a wide range of organisms such as plants, invertebrates and mainly marine vertebrate species^1–10^. Recently, fluorescence was discovered in the South American tree frog *Boana* (formerly *Hypsiboas*) *punctata*, produced by compounds in lymph and skin glands^11^. Little is known about the function or evolution of fluorescence of organisms^11,12^, but conclusions and hypotheses include photo-protection in excessive sunlight^3^, UV-light detection, prey-^4^ or pollinator-attraction^5^, and signalling for mate choice, species recognition, and male-male interaction^2,6,7,9,13^. Proteins, pigments, chitin and lymph/glandular components are known to be the fluorescent agents of these organisms^11,14^.

Chameleons often show remarkable colouration^15,16^ and have conspicuous bony crests and tubercle pattern on their heads. The shape, size and distribution of these crests are taxonomically informative^17^ and sexually dimorphic^18,19^, but their purpose has never been established. It is probable that their function is similar to ornamentations used by other taxa for species recognition and intraspecific signalling and communication^19^. We investigated the properties of these tubercles, and here report the first known instance of externally visible bone-based fluorescence in vertebrates. Bone has long been known to fluoresce under UV light^20^, a phenomenon that has been used in forensic research^21^, but no organism has so far been reported to co-opt this phenomenon for fluorescent signalling. Chameleons are already famed for their exceptional eyes and visual communication^22,23^, and now they are among the first known terrestrial squamates that display and likely use fluorescence.

## Results and Discussion

### Fluorescence of bony tubercles in chameleons

We discovered that the crests and tubercles on the heads of many chameleon species emit blue fluorescence when excited with UV light. Focusing on the genus *Calumma* we investigated the osteological and histological basis of this phenomenon and the sex/species specificity of its patterns, and its phylogenetic distribution across all chameleon genera.

**Figure 1:**
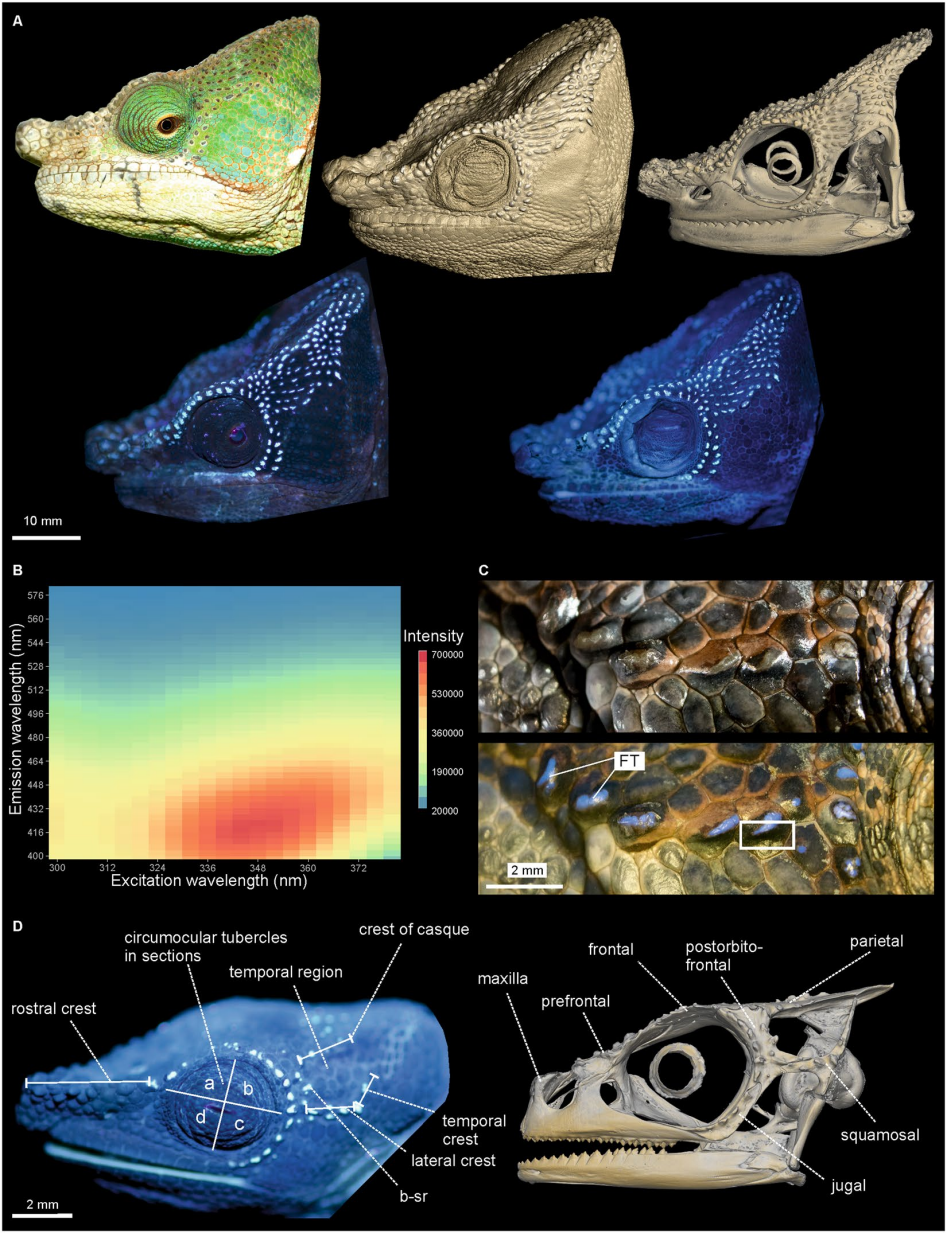
Chameleons of the genus *Calumma* with fluorescent tubercles of bony origin. (**A**) Male *C*. *globifer* (ZSM 141/2016) showing congruent tubercle/fluorescent patterns (from left to right); top row: alive in the field under sunlight, micro-CT scan of head surface (probable edge artefact in cheek region), micro-CT scan of the skull; bottom row: alive in the field under UV light, ethanol-preserved under UV light. (**B**) Excitation-emission matrix (intensity in arbitrary units) of fluorescent tubercles on right temporal region of *C*. *globifer* (ZSM 221/2002). (**C**) Fluorescent tubercles (FTs) on temporal region (right body side) of a male *C*. *crypticum* (ZSM 503/2014) under visible (above) and additional UV light (below); framed area of the skin, including a FT, was histologically sectioned (Fig. 2). (**D**) Distribution of FTs on the head surface (left) and micro-CT scan (right) of the head of *C*. *guibei* (ZSM 2855/2010) shows the bony origin of the FTs.

**Figure 2:**
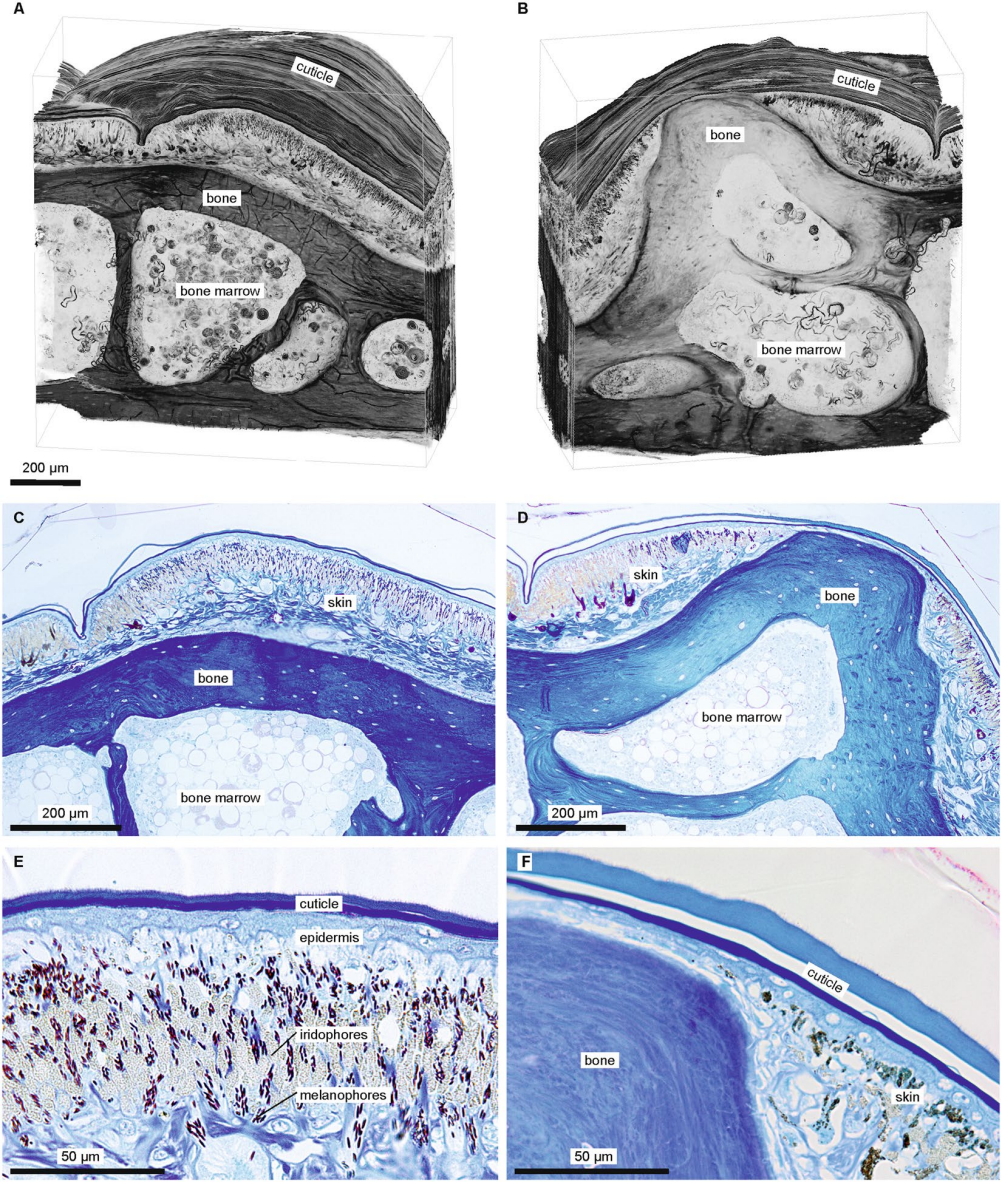
Histological sections of skin of a male *Calumma crypticum* (ZSM 503/2014) from the temporal region (framed in Fig. 1C). (**A,B**) 3D-reconstruction (volume rendering) of 279 histological sections of skin (without tubercle) (**A**) and of tubercle (**B**), i.e. upper margin of frame in Fig. 1C). (**C**) Section of skin and underlying bone in adjacent skin, stained (Richardson). (**D**) Section of FT (centre, stained). (**E**) Detail of skin near FT (stained). (**F**) Detail of FT (stained). For detailed views of the chromatophores, see Supplementary Fig. S5.

Living and ethanol-preserved *Calumma* chameleons exhibit characteristic tubercle patterns of blue fluorescence on their heads (Fig. 1A). The optimal excitation wavelength is in the UV-A spectrum at 353 nm, inducing an emission spectrum from 360 nm to 500 nm, with a maximum at 433 nm (Fig. 1B) measured from *C*. *globifer* and *C*. *crypticum* without notable variation between the species. The fluorescent elements are the centres of raised scales (Fig. 1C) in most cases. Micro-CT scans reveal these raised scales to be caused by tubercular outgrowths of the underlying bones (Fig. 1A,D, Supplementary Figs S1–4). Histological sections of a fluorescent tubercle (FT, Fig. 2) demonstrate that the top of the bony tubercle is only covered by a thin layer (20–25 µm) of epidermis that functions as a ‘window’ through which the bone is directly visible (Fig. 2B,D,F, Supplementary Fig. S5A). The bony tubercle displaces the dermis containing melanophores and chromatophores^24^, which are present around the protuberance and identical to that of a non-tubercle scale (Fig. 2A,C,E), rendering the layer covering the tubercle thin and transparent. A comparison of fluorescence spectra of a FT with the underlying bone showed that the emission peak of the bone covers the same range and is broadened towards longer wavelengths with a maximum at about 445 nm (Supplementary Fig. S5F,G). This indicates that the FT spectrum is included in that of the bone. Obviously the thin layer of epidermis acts as an optical filter and shifts the fluorescence towards a ‘bluer’ emission spectrum.

### Distribution patterns of FTs

**Figure 3:**
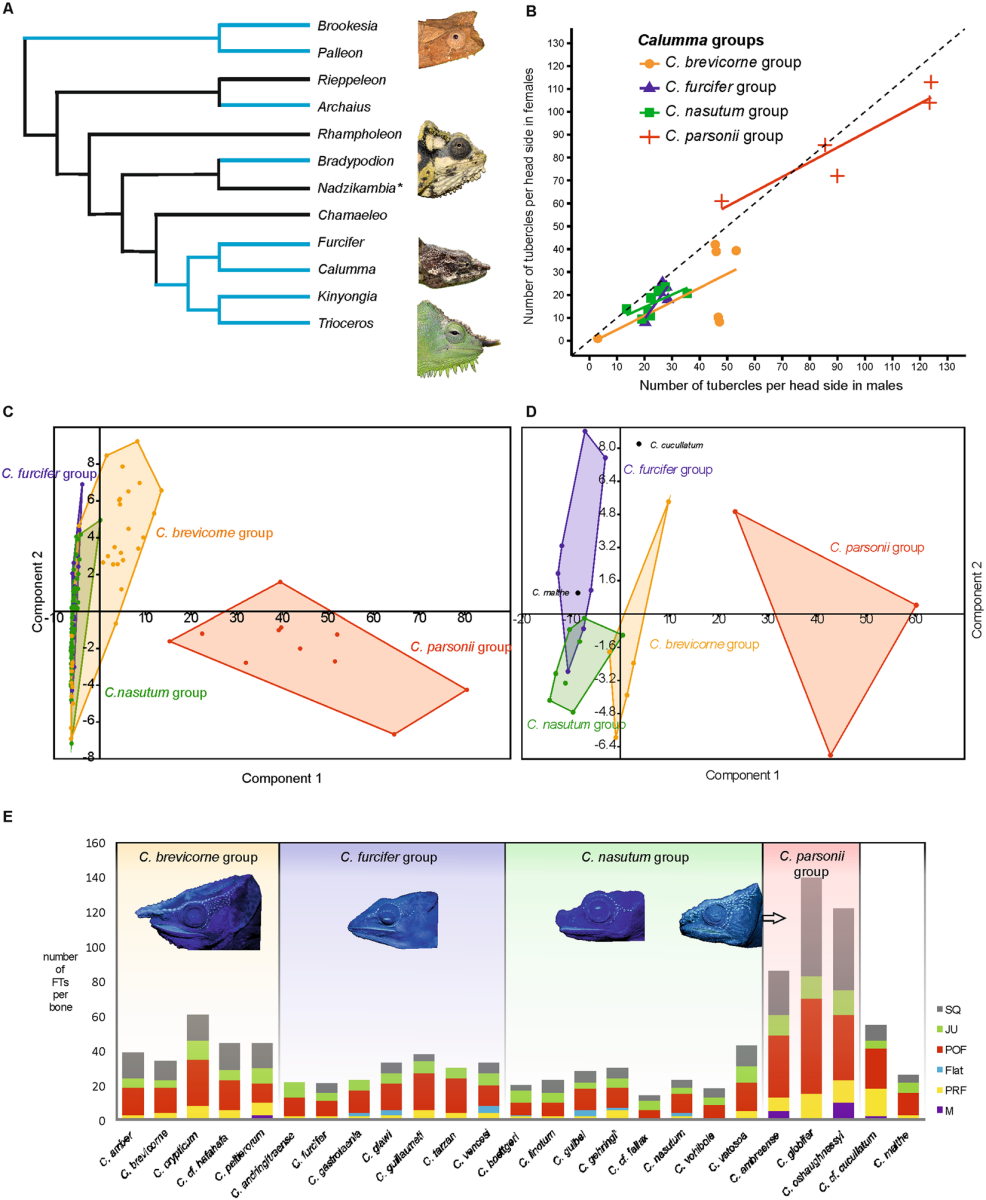
Analysis of distribution of FTs on adult individuals of *Calumma* species groups. (**A**) Schematic chameleon phylogeny of Tolley *et al*.^27^ updated with genus *Palleon*, light blue lines indicate genera where fluorescence occurs (* indicates genus where no data was available). (**B**) Mean value of fluorescent tubercles (FTs) per head side and species, males plotted against females of 126 individuals/24 species assigned to four species groups; dashed diagonal line shows 1:1 ratio, samples below it show more tubercles in males than females. (**C,D**) PCA scatter plots assigned to four species groups, factor loadings are given in Supplementary Tables S2, 3; (**C**) Distribution of FTs per head side based on 12 characters (Supplementary Table S4) of 140 individuals/29 species. (**D**) Number of FTs per cranial bone from lateral view of 25 adult males of different *Calumma* species based on 6 characters (Supplementary Table S5). (**E**) Bar charts of number of FTs per cranial bone (M, maxilla; PRF, prefrontal; Flat, frontal seen laterally; POF, postorbitofrontal; JU, jugal; SQ, squamosal; see Fig. 1D) from lateral view of 25 adult males of different *Calumma* species.

**Figure 4:**
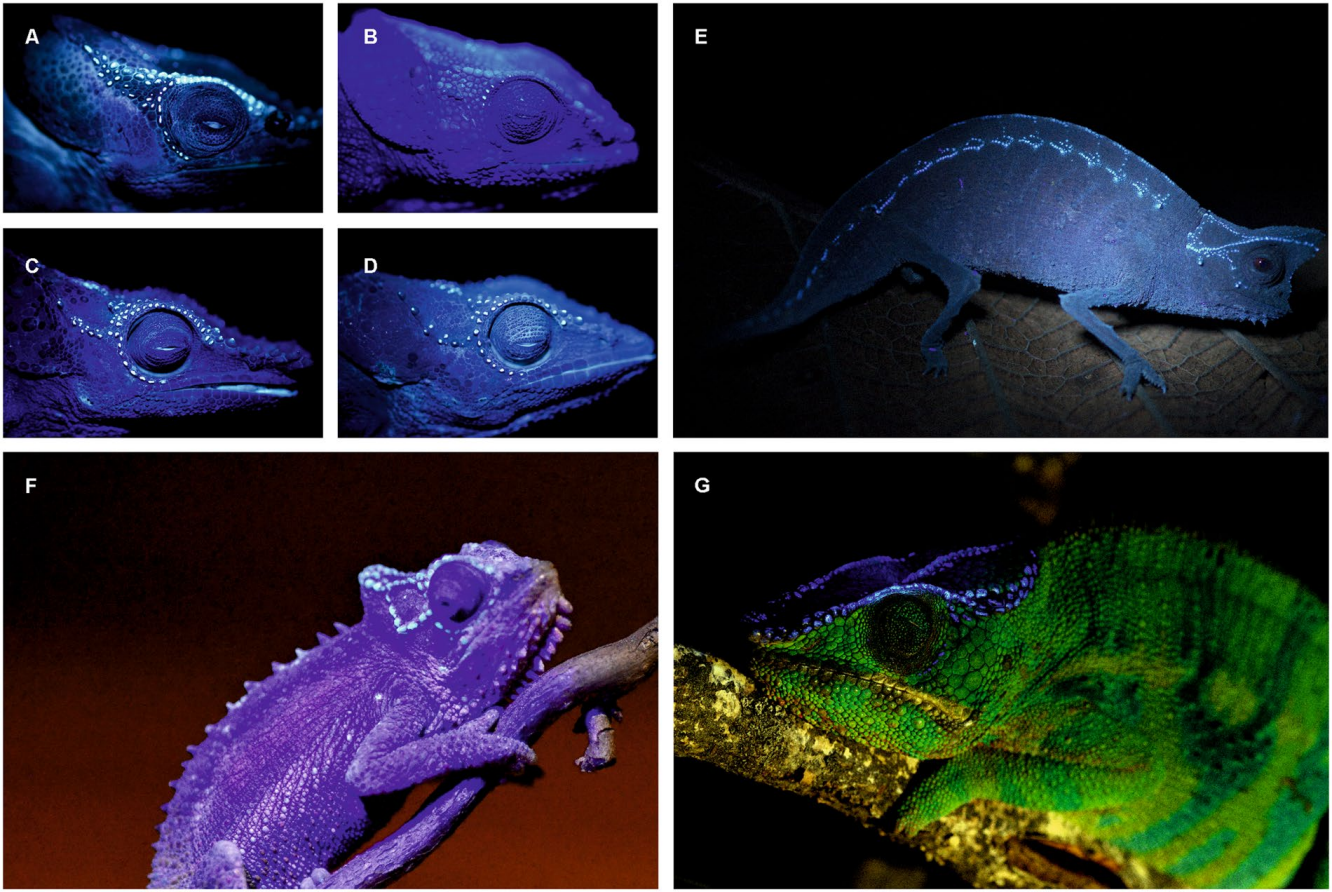
Fluorescent tubercles showing sexual dimorphism under UV light at 365 nm (**A–D**) and fluorescence in further chameleon genera (**E–G**). (**A**) Male *Calumma crypticum* ZSM 32/2016. (**B**) Female *C*. *crypticum* ZSM 67/2005. (**C**) Male *C*. *cucullatum* ZSM 655/2014. (**D**) Female *C*. *cucullatum* ZSM 654/2014. (**E**) *Brookesia superciliaris*, male (only UV light at 365 nm). (**F**) *Bradypodion transvaalense*, male (dim light and additional UV light at 395 nm). (**G**) *Furcifer pardalis*, male (daylight and additional UV light at 365 nm). For details see ‘fluorescent photography’ in Materials and Methods.

This externally visible bone-based fluorescence is not restricted to *Calumma* but also occurs in at least 8 of the 12 chameleon genera currently recognized (Fig. 3A). We focused on the species specificity and sexual dimorphism of *Calumma*. A quantitative analysis of FTs in 24 of the 34 species^25,26^ (126 adult specimens in total) shows that male individuals have on average more tubercles than females in almost all species (Fig. 3B). As we were restricted to using only relatively fresh material, despite having one of the largest collections of *Calumma* specimens outside Madagascar at our disposal, sample sizes per species were low, and the statistical significance of sexual dimorphism is therefore limited; although ANOVA results provide significantly more tubercles in the *C*. *brevicorne* group, with p = 0.016, and *C*. *nasutum* group, with p = 0.003 (Supplementary Table S1). The trend appears to be strong across all species (Figs 3B and 4A–D), and we expect that greater sampling will only strengthen it further.

FTs are concentrated around the eye and the temporal region (Supplementary Figs S1–4), which are important areas for colour signalling among chameleons. Their distribution differs however among species, and even more so among species groups (Fig. 3C). A quantitative comparison of the FTs per cranial bone (Fig. 1D) in males of different *Calumma* species is systematically and phylogenetically informative (Fig. 3D,E). Members of the *C*. *parsonii* group for instance differ clearly from the other species by the presence of a high number of FTs in the temporal region, caused by the broadened postorbitofrontal and squamosal bone which are fused and are densely covered with tubercles (Supplementary Fig. S4). The differences in FT patterns among closely related species are inconsistent, with some sister-species pairs (e.g. *C*. *brevicorne* and *C*. *crypticum*^27^) having strongly overlapping values, while others (e.g. *C*. *tsaratananaense* and *C*. *hafahafa*) are distinctly different (Supplementary Table S4). FT patterns strongly reflect species groups, and are therefore of significant systematic value at the supraspecific level within *Calumma* (Fig. 3E), but their role within species groups requires further study.

### Hypothesis of ecological relevance of fluorescence in chameleons

Phylogenetically, it seems FTs are plesiomorphic to Chamaeleonidae, but are most widespread in *Calumma* being found in almost all known species (Fig. 3A, Supplementary Table S4). In their sister genus *Furcifer*, by contrast, we found only six of 20 studied species to have FTs (Fig. 4G, Supplementary Table S6). *Furcifer* is typically found in more open and dryer habitats in Western Madagascar whereas *Calumma* are usually found in shady, humid forest habitats^28^. Also in *Brookesia*, which are terrestrial and forest dwelling chameleons from Madagascar, bone-based fluorescence even across the whole body is common (Fig. 4E). As shorter (UV, blue) wavelengths are scattered more strongly than longer wavelengths^29^ the UV component under the diffuse irradiation in the forest shade is relatively higher compared to the direct irradiation by the sunlight. Consequently, using UV reflections for communication is apparently more common in closed habitats than in open habitats, as has been shown in chameleons of the genus *Bradypodion*^30^. Members of the latter genus also show fluorescence in the head region (Fig. 4F). A function of FTs as UV reflectors could therefore be hypothesised. This can be ruled out however, as these structures do not reflect UV light at 365 or 390 nm (Fig. 1B, Supplementary Fig. S6A) and at least some chameleon lenses are only transmissive above 350 nm^31^. On the other hand, the emission spectrum of FTs with a maximum at 433 nm is deep blue, a colour that is reasonably rare in a tropical forest and appears to be a conspicuous signal against the background reflectance of grey brown leaf litter or green vegetation as shown in Fig. 1 of Andersson *et al*.^32^. The glowing blue of the FTs is near to the maximum absorption of the pigments of the SWS (short-wave-sensitive) cones with 440–450 nm of the examined chameleons^31^. Additionally, wavelengths of around 433 nm might appear brighter to chameleons as their visual spectrum is shifted towards shorter wavelengths (from about 350 nm to 650 nm) compared to the human visual perception. Assuming that in the shade of a closed forest canopy the relative intensity of the diffuse UV-light is even higher the fluorescent part of the total reflectance might increase considerably. A quantum yield calculation revealed 0.29% of absorbed photons being emitted as fluorescence. This is a typical fluorescence quantum yield for a dye that is immobilised in a matrix (here the bone) and not in a solution (Schwager unpubl.).

Constant fluorescent patterns potentially give chameleons a secondary, stable signalling system that is not influenced by their well-known communication by colour change, and does not compromise their camouflage. Moreover, the systematic relevance of the FTs suggests that they at least correlate with trends in skull evolution, and they may have provided substrate for sexual selection. On this basis, the distribution of FTs could also be used by taxonomists as an additional character to delimitate between taxa. Sexual dichromatism based on fluorescence has been shown in birds^33^ which are also highly visual animals. The use of chameleons as model organisms in the understanding of the role of visual ecology and behaviour in sexual selection^34,35^ will require some adjustment to account for this newly discovered phenomenon. Important future steps will be behavioural trials as well as the creation of colour visual models (accounting for the spatial acuity of chameleon vision) in order to explore the biological relevance of this phenomenon.

Given the recent discoveries of fluorescence, especially among terrestrial vertebrates^11,36^, it is clear that fluorescence evolved by several independent and unique mechanisms. Apparently this tends to occur by the evolution of new mechanisms, rather than implementing features that already have fluorescent properties. Use of such features would be expected to be the most obvious route to fluorescence. We suspect, however, that chameleons are not unique in this regard, and that bone-based fluorescence is in fact widespread among taxa that use bones in their ornamentation, especially other squamates. Fluorescence in terrestrial vertebrates has been underestimated until now, and its role in the evolution of ornamentation remains largely unexplored, but this is a promising avenue for future research.

## Materials and Methods

### Investigated chameleon specimens

160 specimens covering 31 species (Supplementary Table S4) of the genus *Calumma* from the collection of the Zoologische Staatssammlung München, Germany (ZSM), the Museo Regionale di Scienze Naturali, Torino, Italy (MRSN), and Senckenberg Museum, Frankfurt am Main, Germany (SMF), were photographed and quantitatively analyzed for the presence and distribution of FTs. Only specimens that were collected in 1998 or later were investigated, due to the decay in their fluorescent ability in preservative^21^. The following species were considered: *C*. *amber*, *C*. *brevicorne*, *C*. *crypticum*, *C*. *hafahafa*, *C*. *hilleniusi*, *C*. *peltierorum* and *C*. *tsaratananense* of the *C*. *brevicorne* group^37^; *C*. *andringitraense*, *C*. *furcifer*, *C*. *gastrotaenia*, *C*. *glawi*, *C*. *guillaumeti*, *C*. *tarzan* and *C*. *vencesi* of the *C*. *furcifer* group^38^; *C*. *boettgeri*, *C*. *fallax*, *C*. *gallus*, *C*. *gehringi*^26^, *C*. *guibei*, *C*. *linotum*, *C*. *nasutum*, *C*. *peyrierasi*, *C*. *vatosoa*^39^ and *C*. *vohibola* of the (phenetic) *C*. *nasutum* group^39^; *C*. *ambreense*, *C*. *capuroni*, *C*. *globifer*, *C*. *oshaughnessyi*, *C*. *p*. *parsonii* and *C*. *p*. *cristifer* of the *C*. *parsonii* group^37^; the species *C*. *cucullatum* and *C*. *malthe* are phylogenetically isolated^27^ and were not assigned to a *Calumma* species group. Additionally 20 species/165 specimens (of the 22 known species) of the genus *Furcifer* from the ZSM collection catalogued in the year 1998 or later were checked for fluorescence (Supplementary Table S6). All other chameleon species of the ZSM were checked and exemplarily one fluorescent specimen per genus (if applicable; no specimens available for *Nadzikambia*) is listed in Supplementary Table S6.

### Fluorescent photography

Photographs were taken with a Nikon D5100, using a Tamron SP AF 90 mm f/2.8 Di macro lens. Both living specimens in the field and ethanol-preserved specimens in the laboratory were photographed with standard settings of F/6.3, 0.5 s, and ISO 200. A Tank007-TK-566 flashlight 3 Watt (Tank007 Lighting Inc.), emitting at a maximum of 365 nm, was arranged in a distance of 100 mm from the sample, as the only light source for photography.

Fluorescent photographs of living animals (one each of *Calumma globifer*, *Furcifer pardalis* and *Brookesia superciliaris*) were taken in Madagascar in December 2015/January 2016 and in August 2016. This method was entirely non-invasive and did not disturb the animals.

Detailed photographs of fluorescent tubercles (FTs) were taken using the binocular Olympus SZX12 (Olympus DF Plapo 1XPF objective) and a Sony Nex-5N digital camera (lens replaced by a camera tubus). The investigated specimen was a male *C*. *crypticum* (ZSM 503/2014, Fig. 1C).

### Ultraviolet photography

UV photographs of an ethanol- preserved *Calumma globifer* (ZSM 141/2016, Supplementary Fig. S6) were taken with a modified Canon EOS 750D (internal UV and IR filter removed) and the uncoated, UV light-transmissive lens (Ultra-Achromatic-Takumar 1:4.5/85) with a Baader U-Filter (60 nmHBW/ 320–380 nm) and a setting of F/4.5, 8/5 s, and ISO 100. The photo lamp DLED4.1-UV365 (40 W, 365 nm with a 366 nm UV pass filter B3) at a 45° angle to the chameleon skin was used as the UV light source.

### Fluorescence spectroscopy

Fluorescence excitation-emission spectra were recorded with a Fluorolog (Horiba) fluorimeter (slit width 3 nm). All fluorescence spectra were corrected for the wavelength-dependent output of the Xenon lamp. The intensity of the fluorescence signal shown here represents the detected emission signal divided by the simultaneously measured intensity of the lamp at the same wavelength.

For the comparison of fluorescence spectra of a FT and the underlying bone parts of the skin were removed on the temporal region of a *Calumma crypticum* (ZSM 503/2014) and emission of the externally visible bone was measured (Supplementary Fig. S5F,H).

### Quantum yield calculation

Using the Fluorolog fluorimeter (see above) the following four spectra were recorded with the same spectrometer settings: for the determination of the absorption, two emission spectra of the excitation signal in the range 340–365 nm (max. fluorescence excitation at 353 nm) of an FT region and non- FT region (as background), considering the same irradiation area; and for determination of the fluorescent signal, two emission spectra from 365–680 nm (max. fluorescence emission at 433 nm) of an FT region and non-FT (as background), considering the same irradiation area. The quantum yield is calculated according to

$$\Phi =\frac{{N}_{emission}}{{N}_{absorption}}$$

where N_emission_ is the integrated background-subtracted signal of the emission and N_absorption_ is the integrated background-substracted signal of the excitation range.

### Analysis of fluorescent pattern

The occurrence of FTs was assigned to different cranial crests and other parts of the head. The nomenclature of crests follows largely Nečas^40^ with some adaptions to the genus *Calumma*, see Fig. 1D. The following characters were analysed: CO, number of FTs on circumocular tubercles in sections (a, b, c, d); RC, number of FTs on the rostral crest (tubercle scales from snout tip to CO, not bordering the eye); b–sr, tubercles that are arranged in a second row behind section ‘b’; LC, number of FTs on lateral crest; TC, number of FTs on temporal crest; CC, number of FTs on crest of casque; TR, number of FTs in temporal region; PC, number of FTs on parietal crest; DH, number of FTs on the dorsal head region.

The FTs of 25 adult males of different *Calumma* species were counted per bone of the skull (on the left side and dorsally)—comparing a fluorescent photograph of the head with a micro-CT scan of the skull of the same specimen (Fig. 1D, Supplementary Figs S1–4, and Table S5). FTs of the following bones were counted: M, maxilla; PRF, prefrontal; Flat, FTs that are only laterally seen on frontal; POF, postorbitofrontal; JU, jugal; SQ, squamosal; Pdors, FTs that are seen dorsally on parietal; Fdors, FTs that are seen dorsally on frontal.

For Principal Component Analyses (PCA) the statistical analysis software PAST 3.08^41^ was used. A PCA was performed for 12 counts of FTs of the sections that are visible in lateral view (RC, CO-a, CO-b, CO-b-sr, CO-c, CO-c-sr, CO-d, LC, LC-sr, TC, CC, TR) of 140 individuals/29 species assigned to one of the four groups within the genus *Calumma* (Fig. 3C). A second PCA was performed for 6 counts of FTs on bones that are visible in lateral view (M, PRF, Flat, POF, JU, SQ) from 25 specimens and species (Fig. 3D).

ANOVAs were calculated in R v3.0.3^42^ based on the total number of FTs per head side (average value of right and left head side) of 133 individuals/25 *Calumma* species (Supplementary Table S1). The residuals of all species groups were checked for normality, and no significant deviations were observed. Due to our unbalanced sampling (variable number of specimens available per species), ANOVAs returned marginally different results depending on whether species or sex was given first in the formula, but this did not have any impact on significance results. Only one species, *C*. *capuroni*, has considerably more tubercles in females than in males, but the sample size of this species is low (2 specimens).

### X-ray micro-CT

For skeletal morphology, X-ray micro-computed tomography scans (micro-CT scans) of the head (or full body) of 70 specimens were prepared. Methods followed Prötzel *et al*.^43^, using diamond or standard target, with the scanner set to 130 kV and 80 µA with a timing of 500 ms, and scans consisting of 1800 projections (i.e. 5 images per degree of rotation) or similar settings. For detailed information, see Supplementary Table S7.

### Histology

A FT from the temporal region (Fig. 1C) of a male *Calumma crypticum* (ZSM 503/2014, stored in 70% ethanol) was dissected with a razorblade. The excised FT was washed in 0.1 M phosphate buffer, stained in buffered 1% Osmium tetroxide (OsO_4_) for one hour on ice, dehydrated in a graded acetone series, embedded in epoxy resin^44^, serial sectioned in 279 planes à 1.54 µm using a RMC MT-7000 ultramicrotome with a Diatome Histo Jumbo diamond knife, mounted on glass slides, and partly stained with a 1:1 mixture of methylene blue and Azure II for approx. 5 s at 80 °C^45^. Unstained sections (Supplementary Fig. S5A,B) were sealed under coverslips with DPX mounting medium (Agar Scientifics). The glass slides with slice-ribbons were imaged in bright field illumination using an Olympus BX61VS light microscope with UPlanSApo 10 × NA 0.4 objective and CX10 digital camera, and the program VS-ASW FL (Olympus v 2.7) for virtual slide acquisition. Images of single slices were then extracted with OlyVIA software (Olympus v 2.9; 1267 × 2119 px, 24 bit RGB, 0.98 µm/px) for subsequent volume rendering. In addition selected slices were photographed using an Olympus CX 41 light microscope with a DP25 digital camera (objectives: Olympus Plan C 10 × NA 0.25, Olympus UPlanSApo 40 × NA 0.95, and Olympus UPlanSApo 60 × NA 1.2 W); for details see Supplementary Table S8.

### Volume rendering

The colour image stack from OlyVIA was pre-processed in Adobe® Photoshop® CS6 as follows: converted to 8 bit greyscale, brightness and contrast autoscaled, folds in epoxy resin outside the tissue were cropped and replaced by a uniform white background to get a clear view onto the cuticle in 3D renderings (however, some remaining folds inside the tissue cannot be avoided). The stack of greyscale images was imported in Amira 5.6 (FEI), consecutive images were aligned, ROI cropped resulting in a 3D volume of 1011 × 1092 × 297 voxels (voxel size 0.98 × 0.98 × 1.54 µm^3^), LUT inverted, volume rendering applied, LUT re-inverted, zoomed and rotated in appropriate perspectives to show both sides of the stack with bounding box (Fig. 2A,B), screenshots were saved in JPEG format (8 bit greyscale, 1390 × 1065 px, 1.28 µm/px).

### Transmission Electron microscopy

Subsequent to the semithin section series some ultrathin sections (70 nm) were cut with a diamond knife and mounted on formvar-coated copper slot-grids (Agar Scientific G2500C). The slices were double-stained with 8% uranyl acetate and lead citrate^46^ and imaged by using a FEI Morgagni 268 TEM at 80 kV.

## Electronic supplementary material

supplementary information
