# Supplementary material for: Widespread bone-based fluorescence in chameleons
Source: Sci Rep. 2018 Jan 15;8:698. doi: 10.1038/s41598-017-19070-7 (PMC5768862; doi:10.1038/s41598-017-19070-7)
Supplement: Supplementary file 1 — supplementary information [file 41598_2017_19070_MOESM1_ESM.pdf]

Supplementary Information for

Widespread bone-based fluorescence in chameleons

David Prötzel[1], Martin Heß[2], Mark D. Scherz[1], Martina Schwager[3], Anouk van't Padje[1,4] & Frank Glaw[1*]

[1]Department of Herpetology, Zoologische Staatssammlung München (ZSM-SNSB), Münchhausenstr. 21, 81247 München, Germany

[2]Department Biologie II, Ludwigs-Maximilians-Universität München, Großhaderner Straße 2, 82152 Planegg-Martinsried, Germany

[3]Department of Applied Sciences and Mechatronics, Munich University of Applied Sciences, Lothstr. 34, 80335 München, Germany

[4]Department of Ecological Science, Vrije Universiteit Amsterdam, De Boelelaan 1085, 1081 HV Amsterdam, The Netherlands.

[*]Corresponding author, e-mail: frank.glaw@zsm.mwn.de

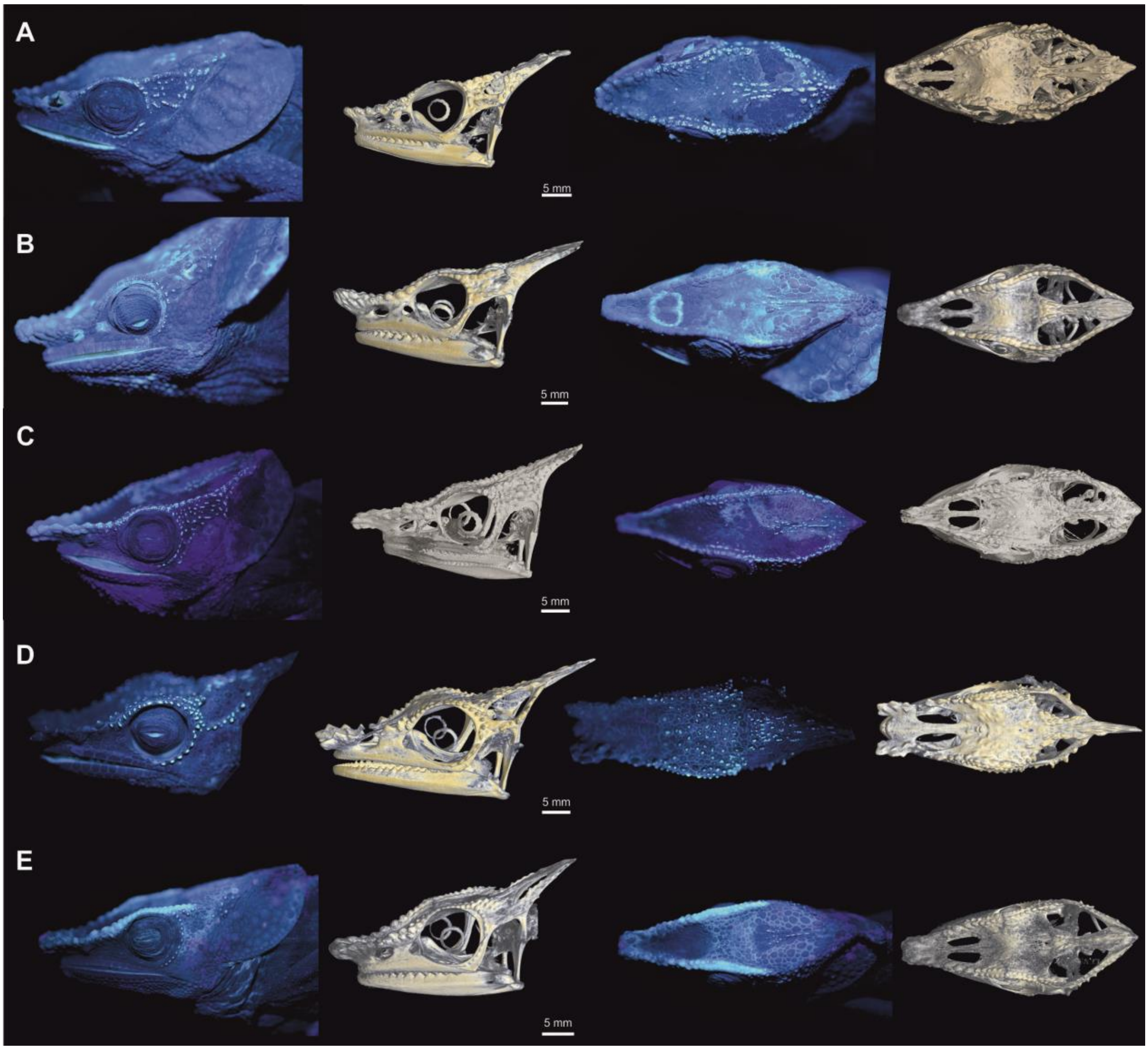

Figure S1. Fluorescence photographs and micro-CT scans of male specimens of the *Calumma brevicorne* group (A–C) and other species (D, E) in lateral and dorsal view. (**A**) *C. amber* ZSM 1041/2003. (**B**) *C. brevicorne* ZSM 549/2001. (**C**) *C. crypticum* ZSM 455/2010. (**D**) *C. cucullatum* ZSM 655/2014. (**E**) *C. malthe* ZSM 71/2005.

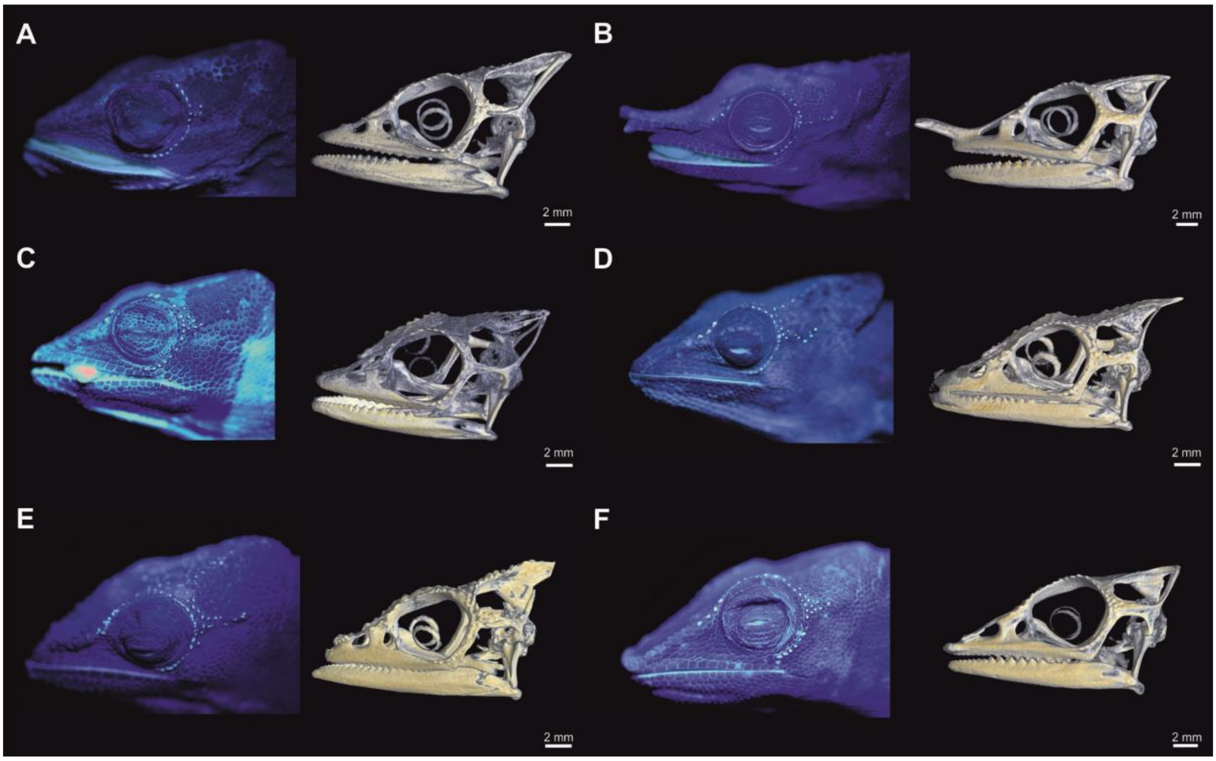

Figure S2. Fluorescence photographs and micro-CT scans of male specimens of the *Calumma furcifer* group in lateral view. (**A**) *C. andringitraense* ZSM 554/2001. (**B**) *C. furcifer* ZSM 225/2002. (**C**) *C. gastrontaenia* ZSM 226/2002. (**D**) *C. glawi* ZSM 2042/2008. (**E**) *C. guillaumeti* ZSM 440/2005. (**F**) *C. tarzan* ZSM 219/2010.

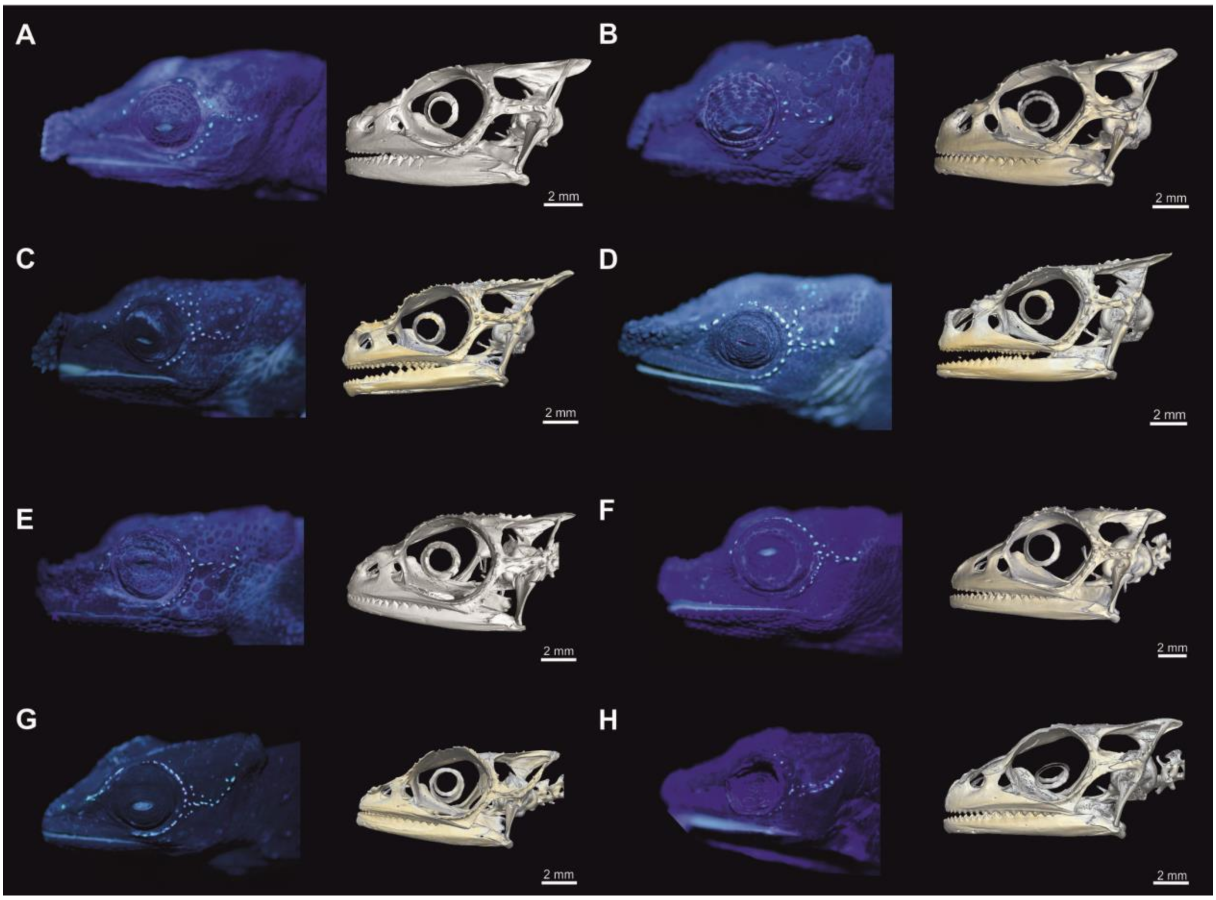

Figure S3. Fluorescence photographs and micro-CT scans of male specimens of *Calumma nasutum* group in lateral view. (**A**) *C. boettgeri* ZSM 440/2000. (**B**) *C.* cf. *fallax* ZSM 693/2009. (**C**) *C. gehringi* ZSM 2851/2010. (**D**) *C. linotum* ZSM 2072/2007. (**E**) *C. guibei* ZSM 2855/2010. (**F**) *C. nasutum* ZSM 618/2009. (**G**) *C. vatosoa* MRSN R1628. (**H**) *C. vohibola* ZSM 645/2009.

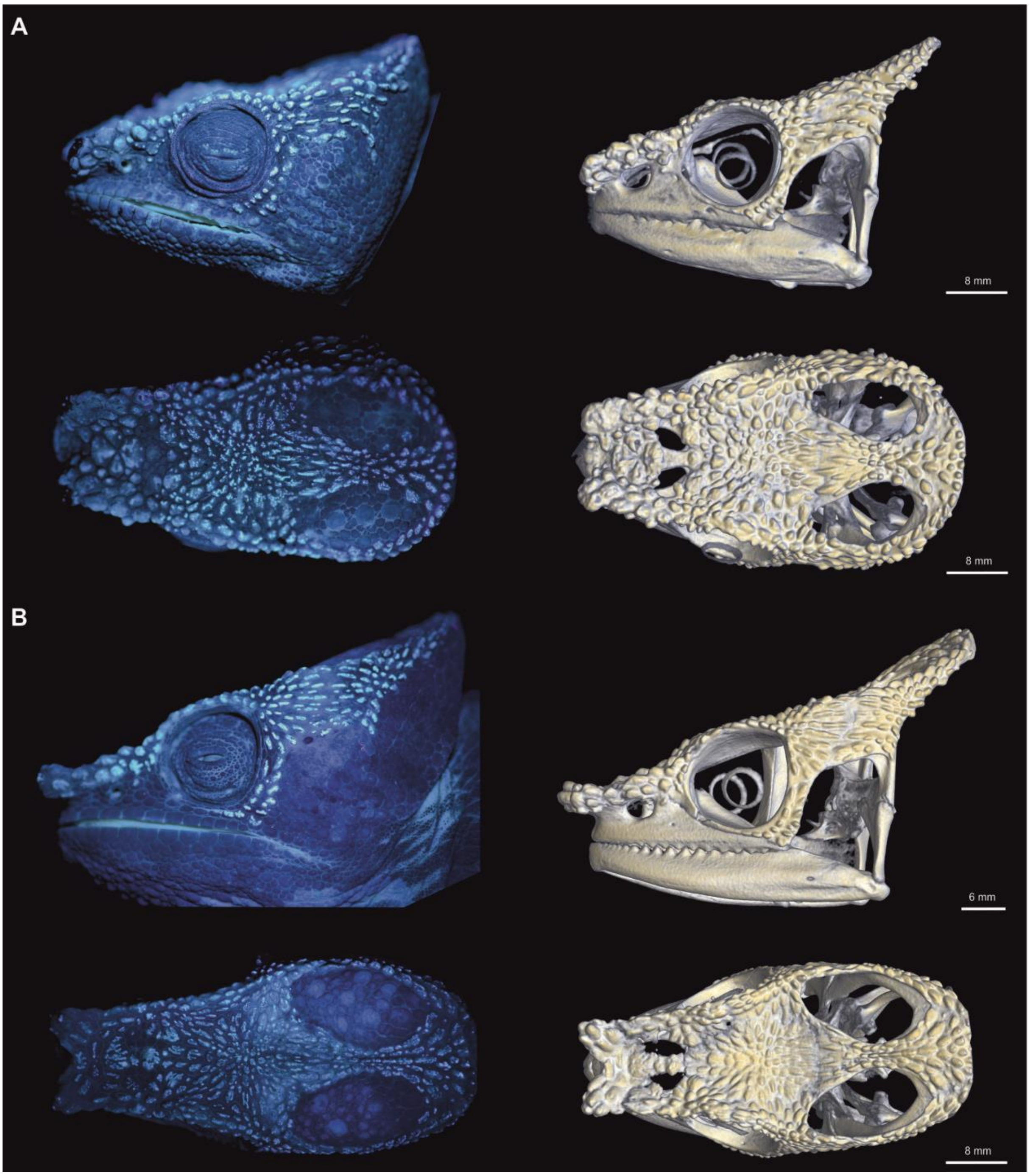

Figure S4. Fluorescent photographs and micro-CT scans of male specimens of *Calumma parsonii* group in lateral and dorsal view. (**A**) *C. ambreense* ZSM 1042/2003. (**B**) *C. oshaughnessyi* ZSM 1836/2010.

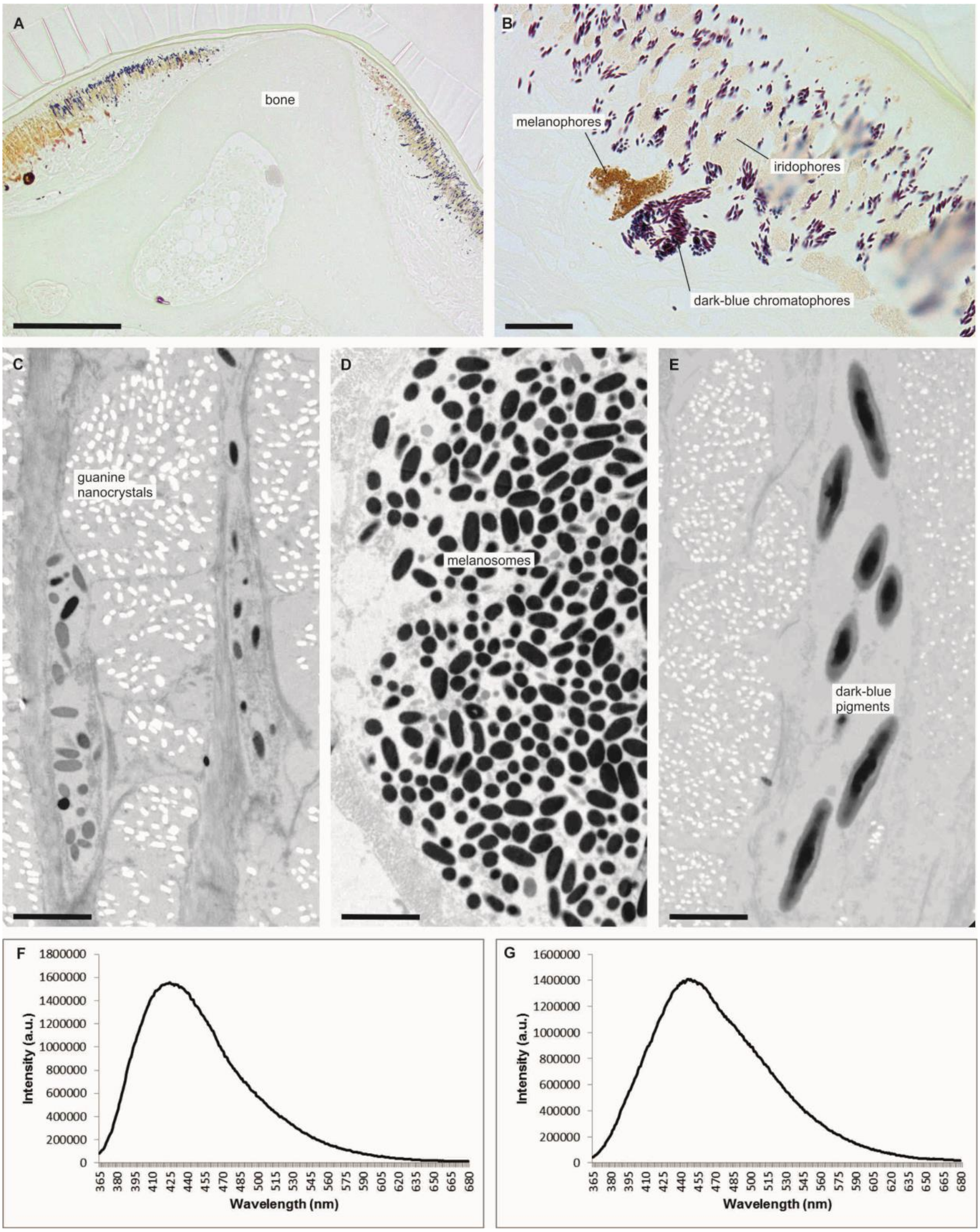

Figure S5. Chromatophores in the skin of a male *Calumma crypticum* (ZSM 503/2014) from the temporal region. (**A**) Semithin section (1.54 µm) of a FT, unstained, 10x. (**B**) Details of skin including 3 types of chromatophores in the dermis, unstained, 60x; (C–E) TEM photographs of the different chromatophores, shown in (B). (**C**) Guanine nanocrystals in iridophores. (**D**) Melanosomes in brown melanophores (up to 450 nm in diameter and 1 µm in length, homogenous material). (**E**) Unknown pigments in dark-blue chromatophores; up to 750 nm in diameter and 3.2 µm in length, electron dense core, less electron dense coat ca. 150 nm). (**F**) Fluorescence spectrum of FT on temporal region of *C. crypticum* (see above) with

emission peak at 425 nm. (**G**) Fluorescence spectrum of underlying bone on temporal region of the same specimen with emission peak at around 445 nm. Scale bars in (A) 200 µm, in (B) 20 µm, in (C–E) 2 µm.

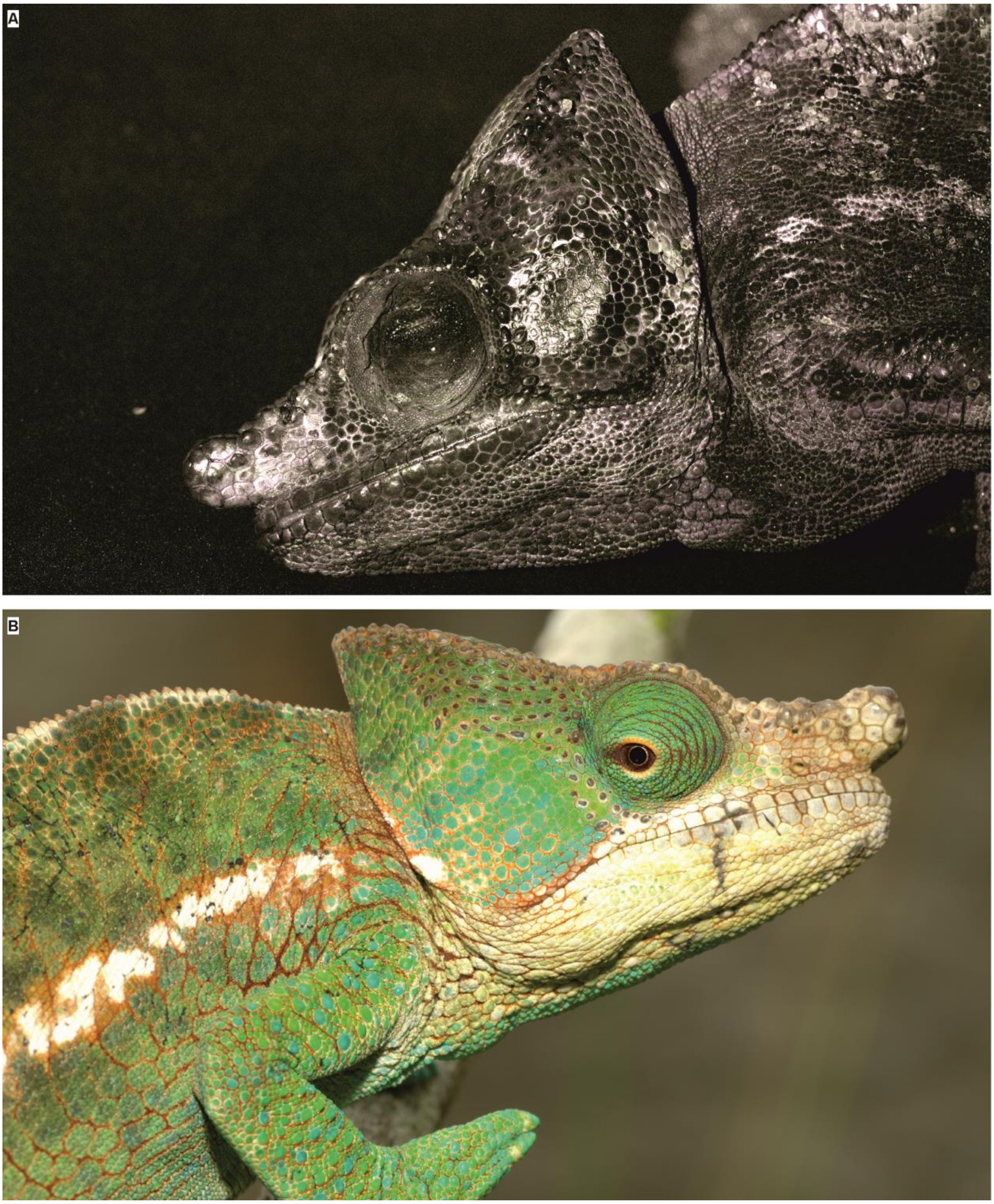

Figure S6. (A) UV photograph of *Calumma globifer*, male (ZSM 141/2016, only UV light at 365 nm, for details, see chapter UV photography; photograph taken by Roy Hessing). (**B**) Photograph of the same specimen under sunlight in the natural habitat.

Table S1. ANOVA results for species- and sex-specificity based on the total number of FTs per head side of 133 individuals/25 *Calumma* species assigned to four species groups. For graphical representation of the extent of sexual dimorphism see Fig. 3*B*. (Df, degrees of freedom; sq, square; *P*, Probability; Significance: '**' < 0.01, '*' < 0.05.

		Df	Sum Sq	Mean Sq	F value	*P*	Significance
Calumma brevicorne species group, response variable (n tubercles), ANOVA anova(lm(tub~spec + sex))	Species	5	3452.2	690.43	3.627	0.016036	*
	Sex	1	2758.3	2758.27	14.490	0.001031	**
	Residuals	21	3997.6	190.36			
Calumma furcifer species group, response variable (n tubercles), ANOVA anova(lm(tub~spec + sex))	Species	4	263.32	65.830	1.3737	0.2803	
	Sex	1	140.51	140.514	2.9321	0.1031	
	Residuals	19	910.53	47.922			
Calumma nasutum species group, response variable (n tubercles), ANOVA anova(lm(tub~spec + sex))	Species	7	1398.13	199.73	3.6115	0.002801	**
	Sex	1	529.94	529.94	9.5820	0.003066	**
	Residuals	56	3097.09	55.31			
Calumma parsonii species group, response variable (n tubercles), ANOVA anova(lm(tub~spec + sex))	Species	4	6462.3	1615.59	6.9377	0.0284	*
	Sex	1	0.9	0.94	0.0040	0.9518	
	Residuals	5	1164.4	232.87			

Table S2. Sex and species specific fluorescent pattern of adult individuals (140 individuals, 29 species). Factor loadings of PC 1 to PC 3 complementary to Fig. 3*C*.

	PC 1	PC 2	PC 3
**RC**	0.087203	0.19697	-0.017932
**a**	0.10219	0.29573	0.095006
**b**	0.058863	0.34484	0.27247
**b-sr**	-0.031373	0.11016	0.42523
**c**	0.036192	0.37266	0.54059
**c-sr**	0.095217	0.086056	0.0049441
**d**	0.00067786	-0.007235	-0.0031641
**LC**	-0.076625	0.24938	0.17161
**LC-sr**	-0.019371	0.065071	0.25557
**TC**	0.0021107	0.38367	-0.33088
**CC**	0.2678	0.57305	-0.47357
**TR**	0.94295	-0.23313	0.12168
**Eigenvalue**	208.589	10.575	5.7124
**% variance**	87.938	4.4583	2.4083

Table S3. Number of fluorescent tubercles per bone of 25 adult males of different *Calumma* species. Factor loadings of PC 1 to PC 3 complementary to Fig. 3*D*.

	**PC 1**	**PC 2**	**PC 3**
**M**	0.072493	-0.10216	0.41616
**PRF**	0.19456	0.26981	0.85568
**Flat**	-0.019186	-0.0059612	-0.07562
**POF**	0.60942	0.71193	-0.29435
**JU**	0.12935	0.025077	0.036106
**SQ**	7.54E-01	-6.40E-01	-3.10E-02
**Eigenvalue**	315.87	18.7527	6.11386
**% variance**	90.758	5.3881	1.7567

Table S4. FTs per head side based on 12 characters of 140 individuals/29 *Calumma* species. See ‘Methods’ for abbreviations.

**species**	**collection number**	**sex**	**head side**	**RC**	**CO**						**LC**	**LC-sr**	**TC**	**CC**	**TR**	**PC**	**DH**
					**a**	**b**	**b-sr**	**c**	**c-sr**	**d**							
***Calumma brevicorne* group**																	
Calumma amber	ZSM 1040/2003	m	left side	5	4	6	0	12	7	0	4	0	5	10	8	8	20
			right side	5	4	7	0	7	3	0	5	0	6	8	7	0	0
Calumma amber	ZSM 1041/2003	m	left side	2	3	5	0	4	0	0	4	0	5	7	9	5	15
			right side	3	3	4	0	5	0	0	4	0	4	7	5	0	0
Calumma amber	ZSM 261/2004	f	left side	0	0	2	0	3	0	0	3	0	0	0	0	0	0
			right side	0	0	-	0	-	0	0	-	0	0	0	0	0	0
Calumma amber	ZSM 260/2004	f	left side	0	0	4	0	3	0	0	4	0	3	0	0	0	0
			right side	0	0	4	0	3	0	0	2	0	2	0	0	0	0
Calumma brevicorne	ZSM 30/2016	m	left side	2	4	6	0	6	0	0	5	0	4	10	7	6	20
			right side	2	4	7	0	8	0	0	4	0	5	10	8	0	0
Calumma brevicorne	ZSM 549/2001	m	left side	0	0	0	0	0	0	0	0	0	0	0	0	0	0
			right side	0	0	0	0	0	0	0	0	0	0	0	0	0	0
Calumma brevicorne	ZSM 1043/2003	m	left side	2	3	7	0	7	0	0	4	0	5	11	15	10	20
			right side	2	4	6	0	8	0	0	4	0	5	12	15	0	0
Calumma brevicorne	ZSM 549/2001	m	left side	1	3	6	0	5	0	0	4	0	6	4	5	7	10
			right side	1	3	6	0	9	0	0	5	0	4	4	6	0	0
Calumma brevicorne	ZSM 31/2016	f	left side	2	1	8	0	5	0	0	4	0	6	5	8	6	10
			right side	2	3	8	0	5	0	0	4	0	5	7	11	0	0
Calumma brevicorne	ZSM 802/2003	f	left side	0	0	0	0	0	0	0	0	0	0	0	0	0	0
			right side	0	0	0	0	0	0	0	0	0	0	0	0	0	0
Calumma brevicorne	ZSM 1684/2012	f	left side	2	2	6	0	7	0	0	4	0	6	7	10	5	15
			right side	2	2	6	0	8	0	0	4	0	5	5	10	0	0
Calumma crypticum	ZSM 32/2016	m	left side	4	5	8	0	7	0	0	3	0	5	7	5	7	15
			right side	3	5	11	0	7	0	0	4	0	4	8	10	0	0
Calumma crypticum	ZSM 135/2016	m	left side	8	6	6	0	7	6	0	5	0	4	11	10	10	30
			right side	6	5	7	0	9	7	0	4	0	4	14	9	0	0

Calumma crypticum	ZSM 1022/2003 1044?	m	left side	1	3	6	0	5	0	0	5	0	6	8	13	7	15
			right side	0	5	5	0	7	0	0	5	0	7	10	14	0	0
Calumma crypticum	ZSM 68/2005	m	left side	0	0	3	0	2	0	0	3	0	2	0	0	3	4
			right side	0	0	3	0	2	0	0	3	0	2	0	0	0	0
Calumma crypticum	ZSM 455/2010	m	left side	3	6	6	0	6	5	0	3	0	5	12	15	5	20
			right side	3	5	7	0	8	0	0	5	0	7	12	17	0	0
Calumma crypticum	ZSM 1711/2010	m	left side	1	6	6	0	7	2	0	5	0	5	9	8	4	30
			right side	2	5	7	0	4	0	0	5	0	4	10	7	0	0
Calumma crypticum	ZSM 1712/2010	m	left side	2	5	7	0	7	4	0	5	0	4	11	10	9	40
			right side	3	3	5	0	7	3	0	6	0	5	10	8	0	0
Calumma crypticum	ZSM 1713/2010	m	left side	3	4	6	0	6	3	0	6	0	5	10	12	7	40
			right side	3	5	8	0	8	2	0	7	0	4	12	11	0	0
Calumma crypticum	ZSM 503/2014	m	left side	2	3	8	0	7	0	0	5	0	6	2	0	5	20
			right side	2	3	7	0	8	0	0	4	0	6	4	0	0	0
Calumma crypticum	ZSM 502/2014	jm	left side	0	0	3	0	6	0	0	5	0	4	0	7	4	4
			right side	0	0	4	0	6	0	0	4	0	5	0	1	0	0
Calumma crypticum	ZSM 1723/2010	jm	left side	0	0	3	0	4	0	0	4	0	3	0	0	6	10
			right side	0	0	3	0	5	0	0	4	0	3	0	3	0	0
Calumma crypticum	ZSM 67/2005	f	left side	0	5	0	0	0	0	0	3	0	1	0	0	0	4
			right side	0	5	0	0	0	0	0	1	0	0	0	0	0	0
Calumma crypticum	ZSM 133/2005	f	left side	0	0	4	0	3	0	0	0	0	0	0	0	0	0
			right side	0	0	3	0	4	0	0	3	0	1	0	0	0	0
Calumma crypticum	ZSM 504/2014	jf	left side	2	5	7	0	8	0	0	5	0	6	7	5	7	15
			right side	3	4	7	0	7	0	0	5	0	5	7	3	0	0
Calumma crypticum	ZSM 617/2009	m	left side	3	6	7	0	3	0	0	4	0	4	13	4	8	15
			right side	3	5	8	0	8	1	0	5	0	4	11	3	0	0
Calumma hafahafa	ZSM 511/2014	f	left side	0	6	6	0	7	0	0	4	0	7	3	7	8	20
			right side	0	7	7	0	7	0	0	3	0	5	3	6	0	0
Calumma hilleniusi	ZSM 315/2006	m	left side	0	0	0	0	0	0	0	0	0	0	0	0	0	0
			right side	0	0	0	0	0	0	0	0	0	0	0	0	0	0
Calumma hilleniusi	ZSM 380/2000	m	left side	0	0	0	0	0	0	0	0	0	0	0	0	0	0
			right side	0	0	0	0	0	0	0	0	0	0	0	0	0	0
Calumma hilleniusi	ZSM 593/1998	f	left side	0	4	6	0	4	0	0	5	0	4	8	6	7	30
			right side	0	3	8	0	4	0	0	4	0	3	9	8	0	0
Calumma hilleniusi	ZSM 594/1998	f	left side	0	5	6	0	6	0	0	5	0	3	7	8	0	0
			right side	0	3	8	0	4	0	0	4	0	3	9	9	0	0
Calumma hilleniusi	ZSM 545/2001	f	left side	0	0	0	0	0	0	0	0	0	0	0	0	0	0
			right side	0	0	0	0	0	0	0	0	0	0	0	0	0	0
Calumma peltierorum	ZSM 1725/2010	m	left side	4	5	4	0	7	0	0	3	0	7	6	8	7	50
			right side	4	6	6	0	6	3	0	4	0	7	7	12	0	0
Calumma peltierorum	ZSM 518/2014	m	left side	6	3	4	0	9	7	0	7	1	5	4	13	8	50
			right side	6	3	5	0	7	4	0	6	2	3	7	12	0	0
Calumma peltierorum	ZSM 519/2014	f	left side	2	0	5	0	5	1	0	4	0	5	3	8	7	50
			right side	2	0	3	0	7	1	0	4	2	4	3	10	0	0
Calumma peltierorum	ZSM 520/2014	f	left side	3	0	7	0	7	1	0	6	0	4	6	9	9	15

			right side	2	0	7	0	7	2	0	5	0	4	5	8	0	0
Calumma peltierorum	ZSM 521/2014	f	left side	0	2	4	0	5	4	0	5	0	6	4	8	8	40
			right side	0	2	4	0	9	3	0	7	0	6	3	12	0	0
Calumma peyrierasi	ZSM 522/2014	m	left side	0	0	4	0	5	0	0	4	0	0	0	0	0	0
			right side	0	0	4	1	3	0	0	4	0	0	0	0	0	0
Calumma peyrierasi	ZSM 1726/2010	jm	left side	0	1	3	0	1	0	0	5	0	0	0	0	0	0
			right side	0	1	2	0	4	0	0	4	0	1	0	0	0	0
Calumma peyrierasi	ZSM 1727/2010	f	left side	0	0	4	0	7	0	0	6	0	1	0	0	0	0
			right side	0	1	3	0	7	0	0	6	0	1	0	0	0	0
Calumma peyrierasi	ZSM 523/2014	f	left side	0	0	3	0	7	0	0	2	0	0	0	0	0	0
			right side	0	0	3	0	7	0	0	4	0	0	0	0	0	0
Calumma tsaratananaense	ZSM 524/2014	m	left side	0	0	1	0	0	0	0	2	0	0	0	0	0	0
			right side	0	0	1	0	0	0	0	2	0	0	0	0	0	0
Calumma tsaratananaense	ZSM 1728/2010	f	left side	0	0	0	0	0	0	0	1	0	0	0	0	0	0
			right side	0	0	0	0	0	0	0	1	0	0	0	0	0	0
***Calumma furcifer* group**																	
Calumma andringitraense	ZSM 554/2001	m	left side	0	0	2	0	1	0	0	2	0	0	0	0	0	0
			right side	0	0	4	1	5	1	0	3	0	0	0	0	0	0
Calumma andringitraense	ZSM 128/2005	m	left side	0	3	5	3	10	1	0	4	0	0	0	0	0	7
			right side	0	3	6	2	7	1	0	4	0	0	0	0	0	0
Calumma andringitraense	ZSM 129/2005	m	left side	0	2	6	1	10	0	0	3	0	0	0	0	0	11
			right side	0	2	8	1	12	1	0	3	0	0	0	0	0	0
Calumma andringitraense	ZSM 130/2005	m	left side	0	1	5	5	9	1	0	3	0	0	0	0	0	0
			right side	0	1	6	1	10	0	0	3	0	0	0	0	0	9
Calumma andringitraense	ZSM 131/2005	f	left side	0	0	4	1	1	0	0	2	0	0	0	0	0	0
			right side	0	0	2	1	2	1	0	2	0	0	0	0	0	0
Calumma furcifer	ZSM 656/2014	m	left side	1	2	6	2	9	0	0	6	3	1	0	0	0	>30
			right side	1	3	7	3	9	0	0	7	3	1	0	0	0	0
Calumma furcifer	ZSM 225/2002	m	left side	1	1	6	0	7	0	0	6	0	0	0	0	0	0
			right side	1	1	5	0	5	0	0	6	0	0	0	0	0	0
Calumma furcifer	ZSM 657/2014	f	left side	0	2	4	1	4	0	0	5	3	1	0	0	0	7
			right side	0	2	6	1	5	0	0	5	2	1	0	0	0	0
Calumma furcifer	ZSM 136/2016	f	left side	0	1	4	0	7	0	0	6	1	0	0	0	0	>20
			right side	0	1	5	1	7	0	0	7	2	0	0	0	0	0
Calumma gastrotaenia	ZSM 140/2016	m	left side	0	1	5	0	6	0	0	5	0	0	0	0	0	0
			right side	0	1	4	0	4	0	0	4	0	0	0	0	0	0
Calumma gastrotaenia	ZSM 226/2002	m	left side	0	2	6	3	6	0	0	4	2	0	0	0	0	0
			right side	0	1	7	2	7	0	0	3	1	0	0	0	0	0
Calumma gastrotaenia	ZSM 506/2014	m	left side	1	2	7	5	9	0	0	4	0	0	0	0	0	1
			right side	1	2	6	4	8	0	0	3	0	0	0	0	0	0
Calumma gastrotaenia	ZSM 659/2014	m	left side	1	3	5	5	8	0	0	7	4	1	0	0	0	>40
			right side	1	3	6	5	8	0	0	8	4	2	0	0	0	0
Calumma gastrotaenia	ZSM 508/2014	m	left side	3	7	7	5	4	0	0	3	2	0	2	0	0	10

			right side	2	3	8	6	6	0	0	5	2	0	3	0	0	0
Calumma gastrotaenia	ZSM 507/2014	f	left side	0	2	6	0	8	0	0	4	2	0	0	0	0	0
			right side	0	2	6	1	6	0	0	4	2	0	0	0	0	0
Calumma gastrotaenia	ZSM 509/2014	f	left side	0	2	5	0	9	0	0	3	0	0	0	0	0	0
			right side	0	2	8	0	7	0	0	4	2	0	0	0	0	0
Calumma gastrotaenia	ZSM 658/2014	f	left side	0	3	7	3	8	0	0	7	2	1	0	0	0	0
			right side	0	3	6	6	9	0	0	8	3	1	0	0	0	0
Calumma glawi	ZSM 2042/2008	m	left side	1	2	7	4	7	0	0	5	2	2	3	0	0	10
			right side	1	2	5	2	9	0	0	5	3	1	4	0	0	0
Calumma guillaumeti	ZSM 440/2005	m	left side	0	5	6	4	8	2	0	5	3	0	5	0	0	>20
			right side	0	5	5	4	11	1	0	8	2	0	3	0	0	0
Calumma guillaumeti	ZSM 1701/2012	m	left side	0	2	5	4	7	0	0	5	2	0	0	0	0	0
			right side	0	2	6	3	8	0	0	3	2	0	0	0	0	0
Calumma guillaumeti	ZSM 34/2016	m	left side	1	3	6	2	11	0	0	4	3	0	0	0	0	0
			right side	1	4	5	2	10	0	0	5	1	0	0	0	0	0
Calumma guillaumeti	ZSM 1703/2012	m	left side	0	0	4	4	6	0	0	4	1	0	0	0	0	0
			right side	0	0	4	4	6	0	0	5	1	0	0	0	0	0
Calumma guillaumeti	ZSM 1702/2012	f	left side	0	2	4	2	8	0	0	4	2	0	0	0	0	0
			right side	0	2	4	2	8	0	0	4	2	0	0	0	0	0
Calumma guillaumeti	ZSM 33/2016	f	left side	0	2	5	1	9	0	0	3	2	0	0	0	0	0
			right side	0	2	7	3	9	0	0	4	3	0	0	0	0	0
Calumma tarzan	ZSM 219/2010	m	left side	2	2	8	4	8	0	0	4	2	0	0	0	0	8
			right side	2	2	7	2	8	0	0	4	2	0	0	0	0	0
Calumma tarzan	ZSM 222/2010	f	left side	0	3	3	1	8	0	0	3	0	0	0	0	0	0
			right side	0	2	4	0	8	0	0	3	1	0	0	0	0	0
Calumma vencesi	ZSM 50/2011	m	left side	2	4	6	3	7	0	0	7	2	2	0	0	0	>40
			right side	3	3	7	4	8	0	0	8	4	2	0	0	0	0
Calumma vencesi	ZSM 462/2010	m	left side	0	0	4	3	7	0	0	5	1	0	0	0	0	>30
			right side	0	0	5	2	4	0	0	5	1	0	0	0	0	0
Calumma vencesi	ZSM 620/2009	m	left side	1	3	7	7	9	0	0	6	6	0	7	0	0	>50
			right side	1	4	7	5	9	0	0	8	5	0	8	0	0	0
***Calumm nasutum* group**																	
Calumma boettgeri	ZSM 440/2000	m	left side	0	2	5	0	7	0	0	6	0	0	0	0	0	0
			right side	0	3	5	0	5	0	0	5	0	0	0	0	0	0
Calumma boettgeri	ZSM 444/2000	m	left side	0	1	4	0	6	0	0	6	0	0	0	0	0	0
			right side	0	2	6	0	6	0	0	7	0	0	0	0	0	0
Calumma boettgeri	ZSM 227/2002	f	left side	0	0	3	0	2	0	0	2	0	0	0	0	0	0
			right side	0	0	5	0	4	0	0	3	0	0	0	0	0	0
Calumma fallax	ZSM 691/2009	f	left side	0	0	0	0	0	0	0	0	0	0	0	0	0	0
			right side	0	0	0	0	0	0	0	0	0	0	0	0	0	0
Calumma gallus	ZSM 137/2016	m	left side	2	3	8	1	5	0	0	6	0	0	2	0	0	10
			right side	2	4	7	1	6	0	0	5	1	1	2	0	0	0
Calumma gallus	ZSM 139/2018	m	left side	1	4	6	0	7	0	0	7	0	0	0	0	0	0
			right side	2	5	9	0	7	0	0	6	0	0	0	0	0	0

Calumma gallus	ZSM 321/2000	m	left side	0	0	3	0	2	0	0	7	2	1	0	0	0	0
			right side	0	1	8	0	5	0	0	5	0	1	0	0	0	0
Calumma gallus	ZSM 792/2003	m	left side	3	2	7	0	6	0	0	6	0	3	0	0	0	0
			right side	3	3	6	0	6	0	0	5	0	3	0	0	0	0
Calumma gallus	ZSM 138/2016	f	left side	0	2	7	1	7	0	0	6	1	0	0	0	0	4
			right side	0	2	8	0	7	0	0	6	0	0	0	0	0	0
Calumma gehringi	ZSM 2840/2010	m	left side	0	2	8	3	3	0	0	5	3	3	0	0	4	>20
			right side	0	4	8	0	5	0	0	5	4	2	0	0	0	0
Calumma gehringi	ZSM 2841/2010	m	left side	0	1	7	0	3	0	0	4	0	0	0	0	0	15
			right side	0	2	6	1	5	0	0	4	0	0	0	0	0	0
Calumma gehringi	ZSM 2851/2010	m	left side	0	4	5	5	6	0	0	6	2	2	0	0	0	0
			right side	0	5	5	4	7	0	0	5	2	2	0	0	0	0
Calumma gehringi	ZSM 2842/2010	m	left side	0	3	7	1	3	0	0	3	0	1	3	0	0	15
			right side	0	3	4	0	4	1	0	4	0	2	4	0	0	0
Calumma gehringi	ZSM 1835/2010	m	left side	0	3	5	0	2	0	0	4	0	0	5	0	0	20
			right side	1	3	6	0	3	0	0	5	0	2	2	0	0	0
Calumma gehringi	ZSM 1834/2010	m	left side	0	1	5	2	2	0	0	4	0	0	0	0	0	0
			right side	1	2	7	2	3	0	0	5	2	0	0	0	0	0
Calumma gehringi	ZSM 2843/2010	m	left side	2	3	7	4	5	2	0	5	0	3	4	0	4	>30
			right side	3	4	7	4	7	0	0	7	0	3	2	0	0	0
Calumma gehringi	ZSM 42/2016	m	left side	0	2	5	3	6	0	0	4	0	0	0	0	4	10
			right side	0	3	6	4	3	0	0	5	0	0	0	0	0	0
Calumma gehringi	ZSM 39/2016	m	left side	3	3	4	4	7	0	0	6	2	4	0	0	0	15
			right side	3	3	6	6	7	0	0	5	3	4	0	0	0	0
Calumma gehringi	ZSM 2844/2010	f	left side	0	2	4	4	5	0	0	5	2	0	0	0	4	20
			right side	0	3	7	5	5	0	0	6	2	0	0	0	0	0
Calumma gehringi	ZSM 2847/2010	f	left side	0	0	6	4	4	0	0	5	3	0	3	0	0	0
			right side	0	0	6	3	4	0	0	5	2	0	0	0	0	0
Calumma gehringi	ZSM 2852/2010	f	left side	0	2	5	3	5	0	0	2	0	1	0	0	0	0
			right side	0	3	4	4	5	0	0	5	3	2	4	0	0	0
Calumma gehringi	ZSM 2845/2010	f	left side	0	2	6	3	5	0	0	5	0	0	5	0	4	20
			right side	0	1	6	4	5	0	0	6	0	1	5	0	0	0
Calumma gehringi	ZSM 2848/2010	jf	left side	0	0	6	2	4	0	0	5	0	0	0	0	0	0
			right side	0	0	4	2	5	0	0	5	0	0	0	0	0	0
Calumma gehringi	ZSM 38/2016	f	left side	0	2	7	0	5	0	0	4	0	0	2	0	4	0
			right side	0	2	5	0	4	0	0	6	2	0	0	0	0	0
Calumma gehringi	ZSM 40/2016	f	left side	0	0	6	1	3	0	0	4	0	0	0	0	0	0
			right side	0	0	7	1	2	0	0	4	0	0	0	0	0	0
Calumma gehringi	ZSM 41/2016	f	left side	0	2	6	3	5	0	0	4	0	1	0	0	4	0
			right side	0	2	6	3	4	0	0	4	0	1	0	0	0	0
Calumma guibei	ZSM 2853/2010	m	left side	2	3	6	4	6	2	0	5	0	2	4	0	0	15
			right side	2	3	6	3	6	0	0	5	0	0	4	0	0	0
Calumma guibei	ZSM 2854/2010	m	left side	2	2	7	5	5	3	0	5	0	2	0	0	5	15
			right side	2	2	8	4	5	3	0	6	0	0	0	0	0	0
Calumma guibei	ZSM 2857/2010	f	left side	0	2	4	3	4	0	0	5	0	0	3	0	2	0

			right side	0	0	6	4	3	0	0	5	0	1	0	0	0	0
Calumma guibei	ZSM 2856/2010	f	left side	0	0	7	3	4	1	0	5	2	1	3	0	4	0
			right side	0	0	6	4	5	0	0	6	1	0	2	0	0	0
Calumma guibei	ZSM 2855/2010	m	left side	0	4	5	4	5	0	0	5	3	2	0	0	4	10
			right side	0	3	5	3	6	0	0	5	3	2	0	0	0	0
Calumma guibei (cf.)	ZSM 1688/2012	m	left side	4	3	7	4	5	0	0	6	4	3	5	5	4	>30
			right side	4	3	8	4	7	4	0	8	5	3	5	4	0	0
Calumma guibei (cf.)	ZSM 1686/2012	jm	left side	0	0	3	0	5	0	0	6	3	1	0	0	0	0
			right side	0	0	4	0	3	0	0	6	2	1	0	0	0	0
Calumma guibei (cf.)	ZSM 1685/2012	j	left side	0	0	4	0	5	0	0	6	1	0	0	0	1	0
			right side	0	0	4	0	5	0	0	7	0	0	0	0	0	0
Calumma guibei (cf.)	ZSM 1687/2012	f	left side	0	0	4	0	4	0	0	7	1	0	0	0	0	0
			right side	0	0	5	0	5	0	0	7	1	0	0	0	0	0
Calumma guibei (cf.)	ZSM 2948/2010	m	left side	5	2	5	4	4	2	0	7	0	1	5	0	4	>40
			right side	5	2	5	4	9	2	0	7	0	1	5	0	0	0
Calumma linotum	ZSM 1683/2012	m	left side	0	2	4	0	0	0	0	6	4	0	0	0	0	0
			right side	0	2	4	0	2	0	0	4	2	0	0	0	3	10
Calumma linotum	ZSM 2072/2007	m	left side	0	2	3	0	7	0	0	5	3	3	0	0	4	8
			right side	0	2	4	1	8	0	0	5	3	2	0	0	0	0
Calumma linotum	ZSM 551/2001	f	left side	0	1	4	1	4	0	0	4	0	0	0	0	0	0
			right side	0	1	4	1	4	0	0	5	0	0	0	0	0	0
Calumma linotum cf.	ZSM 43/2016	m	left side	0	3	8	3	4	0	0	7	0	0	0	0	0	20
			right side	1	5	7	4	7	0	0	5	0	0	0	0	0	0
Calumma linotum cf.	ZSM 142/2016	f	left side	0	0	2	0	2	0	0	4	0	1	0	0	0	0
			right side	0	0	3	0	1	0	0	2	0	0	0	0	0	0
Calumma linotum cf.	ZSM 143/2016	f	left side	0	0	2	0	4	0	0	3	0	0	0	0	0	0
			right side	0	0	2	0	5	0	0	5	0	0	0	0	0	0
Calumma nasutum	ZSM 663/2014	m	left side	0	3	7	3	6	0	0	7	3	3	3	0	0	10
			right side	0	3	7	5	5	2	0	6	2	3	2	0	0	0
Calumma nasutum	ZSM 618/ 2009	m	left side	0	2	7	1	5	0	0	5	2	1	0	0	0	0
			right side	0	5	7	2	7	0	0	6	2	1	0	0	0	0
Calumma nasutum	ZSM 661/2014	f	left side	0	0	4	0	6	0	0	5	3	3	0	0	0	0
			right side	0	0	4	0	5	0	0	5	3	3	0	0	0	0
Calumma nasutum	ZSM 662/2014	f	left side	0	0	4	1	5	0	0	4	0	0	0	0	0	0
			right side	0	0	4	1	4	0	0	6	1	1	0	0	0	0
Calumma nasutum	ZSM 553/2001	f	left side	0	0	5	0	7	0	0	6	0	3	0	0	0	0
			right side	0	1	5	0	6	0	0	5	0	3	0	0	0	0
Calumma nasutum	ZSM 693/2009	m	left side	0	0	3	0	6	0	0	5	0	0	0	0	0	0
			right side	0	0	3	0	6	0	0	5	1	1	0	0	0	0
Calumma nasutum	ZSM 694/2003	m	left side	0	1	2	0	5	0	0	5	0	2	0	0	0	0
			right side	0	1	1	0	6	0	0	5	1	2	0	0	0	0
Calumma nasutum	ZSM 134/2005	m	left side	0	0	2	0	5	0	0	5	0	3	0	0	0	0
			right side	0	0	2	0	6	0	0	5	0	3	0	0	0	0
Calumma nasutum	ZSM 924/2003	m	left side	0	2	5	0	6	0	0	4	0	3	0	0	2	0
			right side	0	1	6	0	9	0	0	6	0	2	3	0	0	0

Calumma nasutum	ZSM 441/2005	m	left side	0	2	5	2	4	0	0	5	0	2	4	0	1	0
			right side	0	2	6	3	3	0	0	7	4	3	4	0	0	0
Calumma nasutum	ZSM 443/2005	f	left side	0	2	5	1	4	0	0	5	1	3	5	0	0	0
			right side	0	2	6	0	3	0	0	5	3	0	4	0	0	0
Calumma nasutum	ZSM 619/2009	m	left side	0	0	7	4	6	2	0	6	0	3	0	0	0	0
			right side	0	0	5	2	6	2	0	6	0	2	0	0	0	0
Calumma nasutum	ZSM 146/2016	f	left side	0	0	5	0	4	0	0	4	0	3	0	0	0	0
			right side	0	0	5	0	5	0	0	5	0	3	0	0	0	0
Calumma nasutum	ZSM 147/2016	f	left side	0	0	3	0	8	0	0	7	0	4	0	0	0	0
			right side	0	0	3	0	10	0	0	6	0	3	0	0	0	0
Calumma nasutum	ZSM 148/2016	f	left side	0	2	1	0	6	0	0	4	2	3	0	0	0	0
			right side	0	0	5	2	6	0	0	5	0	3	0	0	0	0
Calumma nasutum	ZSM 149/2016	f	left side	0	0	3	0	4	0	0	5	0	0	0	0	0	0
			right side	0	0	4	0	5	0	0	5	0	0	0	0	0	0
Calumma nasutum	ZSM 145/2016	m	left side	0	0	3	0	5	0	0	5	0	3	0	0	0	0
			right side	0	0	3	0	7	0	0	5	0	2	0	0	0	0
Calumma nasutum	ZSM 645/2009	m	left side	0	0	4	0	4	0	0	5	3	2	0	0	0	0
			right side	0	0	4	0	5	1	0	4	4	3	0	0	0	0
Calumma nasutum	ZSM 144/2016	f	left side	0	0	7	0	5	0	0	5	0	0	0	0	0	0
			right side	0	0	6	0	5	0	0	4	0	0	0	0	0	0
Calumma vatosoa	MRSN R1628	m	left side	1	4	10	3	7	3	0	6	3	6	0	0	0	5
			right side	1	5	6	3	9	2	0	3	2	5	0	0	0	0
Calumma vohibola	ZSM 644/2009	m	left side	0	0	4	1	7	0	0	5	4	1	0	0	0	0
			right side	0	2	4	0	7	0	0	5	5	1	0	0	0	0
Calumma vohibola	ZSM 643/2009	f	left side	0	0	4	0	5	0	0	5	3	0	0	0	0	0
			right side	0	0	3	0	4	0	0	5	3	0	0	0	0	0
Calumma vohibola	ZSM 152/2016	f	left side	0	0	2	0	3	0	0	5	0	0	0	0	0	0
			right side	0	0	3	0	2	0	0	4	0	1	0	0	0	0
***Calumma parsonii* group**																	
Calumma ambreense	ZSM 1042/2003	m	left side	5	6	7	0	8	6	0	0	0	0	15	39	0	100
			right side	3	7	7	0	6	5	0	0	0	0	12	45	0	0
Calumma ambreense	ZSM 1682/2012	f	left side	3	5	7	0	8	6	0	0	0	0	14	39	0	100
			right side	2	4	9	0	7	6	0	0	0	0	15	46	0	0
Calumma capuroni	ZSM 132/2005	m	left side	4	4	6	0	6	4	0	0	0	0	5	19	4	40
			right side	2	4	7	0	7	1	0	0	0	0	7	20	0	0
Calumma capuroni	ZSM 5104/2005	f	left side	4	6	7	0	5	4	0	0	0	0	9	27	5	60
			right side	4	5	8	0	6	5	0	0	0	0	7	25	0	0
Calumma globifer	ZSM 141/2016	m	left side	4	10	8	0	10	7	0	0	0	0	20	81	10	100
			right side	8	8	8	0	8	6	0	0	0	0	21	85	0	0
Calumma globifer	ZSM 683/2003	m	left side	4	11	8	0	7	5	0	0	0	0	15	51	8	100
			right side	5	11	8	0	7	7	0	0	0	0	15	58	0	0
Calumma globifer	ZSM 221/2002	f	left side	4	7	7	0	5	5	0	0	0	0	14	72	9	60
			right side	4	7	8	0	7	6	0	0	0	0	15	65	0	0
Calumma oshaughnessyi	ZSM 1043/2003	f	left side	6	5	9	0	6	4	0	0	0	0	13	48	0	100
			right side	5	4	9	0	7	6	0	0	0	0	14	46	0	0

Calumma oshaughnessyi	ZSM 1047/2003	f	left side	7	8	8	0	7	6	0	0	0	0	14	53	0	0
			right side	7	6	8	0	7	8	0	0	0	0	13	56	0	0
Calumma p. parsoni	ZSM 151/2016	m	left side	9	8	7	0	7	1	0	0	0	0	12	39	0	70
			right side	8	2	6	0	6	1	0	0	0	0	5	33	0	0
Calumma parsonii cristifer	ZSM 664/2014	f	left side	4	6	11	0	8	5	0	0	0	0	15	41	0	76
			right side	3	8	8	0	7	5	0	0	0	0	17	42	0	0
**others**																	
Calumma cucullatum	ZSM 655/2014	m	left side	6	9	9	11	6	0	0	7	2	5	0	0	0	60
			right side	5	10	8	8	8	0	0	5	2	5	0	0	0	0
Calumma cucullatum	ZSM 653/2014	f	left side	2	5	8	4	7	0	0	4	1	4	4	0	0	25
			right side	3	4	8	3	7	0	0	4	1	3	4	0	0	0
Calumma cucullatum	ZSM 654/2014	f	left side	2	4	7	4	6	0	0	3	1	3	5	0	0	30
			right side	2	4	7	3	7	0	0	6	1	4	4	0	0	0
Calumma malthe	ZSM 71/2005	m	left side	0	1	6	0	7	0	0	0	0	0	4	8	4	10
			right side	0	0	7	0	7	0	0	0	0	0	3	6	0	0
Calumma malthe	ZSM 512/2014	m	left side	7	5	13	0	7	0	0	0	0	0	5	36	5	24
			right side	8	6	9	0	7	0	0	0	0	0	5	35	0	0
Calumma malthe	ZSM 513/2014	m	left side	4	6	9	0	7	0	0	0	0	0	5	23	5	14
			right side	7	4	8	0	4	0	0	0	0	0	4	19	0	0
Calumma malthe	ZSM 515/2014	m	left side	13	7	9	0	7	0	0	0	0	0	9	16	8	13
			right side	15	9	12	0	6	0	0	0	0	0	16	18	0	0
Calumma malthe	ZSM 516/2014	m	left side	7	7	8	0	7	2	0	0	0	0	13	24	7	25
			right side	5	6	8	0	5	1	0	0	0	0	8	21	0	0
Calumma malthe	ZSM 1704/2012	m	left side	14	7	11	0	9	4	0	0	0	0	15	39	8	40
			right side	13	10	12	0	9	2	0	0	0	0	16	40	0	0
Calumma malthe	ZSM 1705/2012	f	left side	6	6	12	0	9	4	0	0	0	0	14	58	8	50
			right side	7	8	9	0	7	3	0	0	0	0	12	40	0	0
Calumma malthe	ZSM 1833/2010	f	left side	2	6	9	0	6	0	0	0	0	0	7	16	3	0
			right side	2	6	9	0	4	0	0	0	0	0	3	12	0	0
Calumma malthe	ZSM 514/2014	f	left side	0	0	4	0	6	0	0	0	0	0	6	4	5	7
			right side	0	0	4	0	7	0	0	0	0	0	6	5	0	0

Table S5. Number of FTs per cranial bone from lateral view of 25 adult males of different *Calumma* species. See 'Methods' for abbreviations.

species	collection number	sex	M	PRF	Flat	POF	JU	SQ	Summe lat.	Pdors	Fdors
***Calumma brevicorne* group**											
Calumma amber	ZSM 1041/2003	m	0	3	0	16	5	15	39	20	0
Calumma brevicorne	ZSM 549/2001	m	0	4	0	15	4	11	34	17	0
Calumma crypticum	ZSM 455/2010	m	0	8	0	27	11	15	61	25	0
Calumma hafahafa	ZSM 617/2009	m	0	6	0	17	6	15	44	15	8
Calumma peltierorum	ZSM 1725/2010	m	3	7	0	11	9	14	44	23	34
***Calumma furcifer* group**											
Calumma andringitraense	ZSM 129/2005	m	0	2	0	11	9	0	22	0	0
Calumma furcifer	ZSM 225/2002	m	0	2	0	9	5	5	21	0	0
Calumma gastrotaenia	ZSM 226/2002	m	0	2	2	13	6	0	23	0	0
Calumma glawi	ZSM 2042/2008	m	0	3	3	15	6	6	33	10	0
Calumma guillaumeti	ZSM 440/2005	m	0	6	0	21	7	4	38	20	20
Calumma tarzan	ZSM 219/2010	m	0	4	0	20	6	0	30	8	0
Calumma vencesi	ZSM 50/2011	m	0	4	4	12	7	6	33	30	30
***Calumma nasutum* group**											
Calumma boettgeri	ZSM 440/2000	m	0	2	1	7	7	3	20	0	0
Calumma linotum	ZSM 2072/2007	m	0	2	0	8	6	7	23	8	4
Calumma guibei	ZSM 2855/2010	m	0	2	4	12	4	6	28	14	0
Calumma gehringi	ZSM 2851/2010	m	0	6	1	12	5	6	30	0	0
Calumma fallax	ZSM 693/2009	m	0	0	0	6	5	3	14	0	0
Calumma nasutum	ZSM 618/ 2009	m	0	2	2	11	4	4	23	0	0
Calumma vohibola	ZSM 645/2009	m	0	0	0	9	4	5	18	0	0
Calumma vatosoa	MRSN R1628	m	1	4	0	17	9	12	43	0	0
***Calumma parsonii* group**											
Calumma ambreense	ZSM 1042/2003	m	5	8	0	36	12	25	86	40	60
Calumma oshaughnessyi	1836/2010	m	10	13	0	38	14	47	122	40	60
Calumma globifer	ZSM 141/2016	m	0	15	0	55	13	57	140	40	70
**others**											
Calumma cucullatum	ZSM 655/2014	m	2	16	0	23	5	9	55	20	40
Calumma malthe	ZSM 71/2005	m	0	3	0	13	6	4	26	14	0

Table S6. Presence (+) or absence (–) of fluorescence in chameleon genera checked with exemplarily specimens.

genus	species	collection number	sex	fluorescence
Archaius				+
	Archaius tigris	ZSM 600/2009	male	+
Bradypodion				+
	Bradypodion thamnobates	ZSM 4742/2012	male	+
	Bradypodion damaranum	ZSM 791/2000	male	+
	Bradypodion thamnobates	ZSM 795/2000	male	
Brookesia				+
	Brookesia ambreensis	ZSM 2090/2007	male	+
	Brookesia antakarana	ZSM 1661/2012	male	+
	Brookesia betschi	ZSM 435/2016	male	+
	Brookesia brunoi	ZSM 888/2010	male	+
	Brookesia brygooi	ZSM 80/2006	-	+
	Brookesia decaryi	ZSM 911/2006	-	+
	Brookesia desperata	ZSM 3276/2012	-	-
	Brookesia ebenaui	ZSM 87/2015	-	+
	Brookesia exarmata	ZSM 72/2006	female	-
	Brookesia griveaudi	ZSM 1677/2012	male	+
	Brookesia karchei	ZSM 1659/2012	female	+
	Brookesia lineata	ZSM 27/2016	male	+
	Brookesia minima	ZSM 990/2003	male	-
	Brookesia perarmata	ZSM 17/2006	male	-
	Brookesia peyrierasi	ZSM 252/2016	female	-
	Brookesia ramanantsoai	ZSM 132/2016	male	-
	Brookesia stumpffi	ZSM 1681/2012	male	+
	Brookesia superciliaris	ZSM 253/2016	male	+
	Brookesia thieli	ZSM 1674/2012	female	+
	Brookesia tuberculata	ZSM 792/2009	male	-
	Brookesia vadoni	ZSM 440/2016	male	+
Chamaeleo				-
	Chamaeleo calyptratus	ZSM 464/1998	male	-
	Chamaeleo chamaeleon	ZSM 935/2014	male	-
	Chamaeleo dilepis	ZSM 4347/2012	-	-
	Chamaeleo laevigatus	ZSM 56/1978	-	-
	Chamaeleo monachus	ZSM 13/1920	-	-
	Chamaeleo namaquensis	ZSM 96/1989/2	-	-
	Chamaeleo senegalensis	ZSM 4712/2012	-	-
Furcifer				+
	Furcifer angeli	ZSM 1057/2003	male	+
	Furcifer antimena	ZSM 624/2000	female	-
	Furcifer antimena	ZSM 625/2000	male	-
	Furcifer antimena	ZSM 780/2000	male	-
	Furcifer antimena	ZSM 4381/2012	female	-
	Furcifer antimena	ZSM 4382/2012	female	-
	Furcifer balteatus	ZSM 682/2003	male	-
	Furcifer balteatus	ZSM 795/2003	-	-
	Furcifer bifidus	ZSM 157/2016	female	-
	Furcifer campani	ZSM 537/2001	male	-
	Furcifer campani	ZSM 538/2001	-	-
	Furcifer campani	ZSM 539/2001	-	-
	Furcifer campani	ZSM 1058/2003	female	-
	Furcifer campani	ZSM 1059/2003	male	+
	Furcifer campani	ZSM 72/2005	male	-
	Furcifer campani	ZSM 316/2006	male	+
	Furcifer campani	ZSM 317/2006	male	-

Furcifer campani	ZSM 723/2014	female	+
Furcifer campani	ZSM 724/2014	female	+
Furcifer campani	ZSM 725/2014	female	-
Furcifer campani	ZSM 630/2003	-	-
Furcifer cephalolepis	ZSM 1686/2008	male	-
Furcifer cephalolepis	ZSM 701/2000	male	-
Furcifer cephalolepis	ZSM 702/2000	female	-
Furcifer cephalolepis	ZSM 324/2002	female	+
Furcifer cephalolepis	ZSM 325/2002	female	+
Furcifer cephalolepis	ZSM 326/2002	male	+
Furcifer cephalolepis	ZSM 327/2002	male	+
Furcifer cephalolepis	ZSM 328/2002	female	+
Furcifer labordi	ZSM 875/2000	male	-
Furcifer labordi	ZSM 782/2000	male	-
Furcifer lateralis	ZSM 28/2004	female	-
Furcifer lateralis	ZSM 185/2004	male	-
Furcifer lateralis	ZSM 2043/2008	-	-
Furcifer lateralis	ZSM 836/2014	male	-
Furcifer lateralis	ZSM 649/2000	female	-
Furcifer lateralis	ZSM 541/2001	-	-
Furcifer lateralis	ZSM 542/2001	-	-
Furcifer lateralis	ZSM 543/2001	male	-
Furcifer lateralis	ZSM 636/2003	male	-
Furcifer lateralis	ZSM 785/2003	male	-
Furcifer lateralis	ZSM 786/2003	female	-
Furcifer lateralis	ZSM 783/2006	male	-
Furcifer lateralis	ZSM 916/2006	male	-
Furcifer lateralis	ZSM 917/2006	female	-
Furcifer lateralis	ZSM 68/2009	-	-
Furcifer lateralis	ZSM 69/2009	-	-
Furcifer lateralis	ZSM 726/2014	male	-
Furcifer major	ZSM 224/2002	female	-
Furcifer major	ZSM 936/2003	male	-
Furcifer major	ZSM 937/2003	male	-
Furcifer major	ZSM 938/2003	male	-
Furcifer major	ZSM 41/2004	male	-
Furcifer major	ZSM 175/2004	male	-
Furcifer major	ZSM 195/2004	male	-
Furcifer major	ZSM 73/2005	-	-
Furcifer major	ZSM 194/2004	male	-
Furcifer major	ZSM 783/2000	male	-
Furcifer minor	ZSM 784/2000	male	-
Furcifer minor	ZSM 786/2000	male	-
Furcifer minor	ZSM 787/2000	female	-
Furcifer minor	ZSM 536/2001	male	-
Furcifer minor	ZSM 918/2006	male	-
Furcifer minor	ZSM 919/2006	female	-
Furcifer minor	ZSM 920/2006	female	-
Furcifer nicosiai	ZSM 20/2006	female	-
Furcifer nicosiai	ZSM 51/2006	female	-
Furcifer nicosiai	ZSM 84/2006	male	-
Furcifer oustaleti	ZSM 243/2004	-	-
Furcifer oustaleti	ZSM 1/2006	male	-
Furcifer oustaleti	ZSM 105/2006	female	-
Furcifer oustaleti	ZSM 58/2009	female	-
Furcifer oustaleti	ZSM 59/2009	female	-
Furcifer oustaleti	ZSM 60/2009	female	-
Furcifer oustaleti	ZSM 61/2009	male	-
Furcifer oustaleti	ZSM 665/2014	male	-
Furcifer oustaleti	ZSM 774/2000	female	-
Furcifer pardalis	ZSM 775/2000	female	-
Furcifer pardalis	ZSM 776/2000	female	-
Furcifer pardalis	ZSM 777/2000	female	-

Furcifer pardalis	ZSM 778/2000	female	-
Furcifer pardalis	ZSM 1060/2003	female	-
Furcifer pardalis	ZSM 1061/2003	female	-
Furcifer pardalis	ZSM 2210/2007	female	-
Furcifer pardalis	ZSM 62/2009	-	-
Furcifer pardalis	ZSM 63/2009	male	-
Furcifer pardalis	ZSM 64/2009	-	+
Furcifer pardalis	ZSM 65/2009	-	-
Furcifer pardalis	ZSM 1730/2010	male	-
Furcifer pardalis	ZSM 4379/2012	male	+
Furcifer pardalis	ZSM 4380/2012	-	+
Furcifer pardalis	ZSM 4680/2012	male	+
Furcifer pardalis	ZSM 316/2014	-	-
Furcifer pardalis	ZSM 317/2014	-	-
Furcifer pardalis	ZSM 318/2014	-	-
Furcifer pardalis	ZSM 319/2014	-	-
Furcifer pardalis	ZSM 320/2014	-	-
Furcifer pardalis	ZSM 321/2014	-	-
Furcifer pardalis	ZSM 322/2014	male	-
Furcifer pardalis	ZSM 323/2014	-	-
Furcifer pardalis	ZSM 324/2014	-	-
Furcifer pardalis	ZSM 527/2014	male	+
Furcifer pardalis	ZSM 1016/2000	-	-
Furcifer pardalis	ZSM 1017/2000	-	-
Furcifer petteri	ZSM 1001/2003	female	-
Furcifer petteri	ZSM 1002/2003	male	-
Furcifer petteri	ZSM 254/2004	male	-
Furcifer petteri	ZSM 258/2004	female	-
Furcifer petteri	ZSM 272/2004	male	-
Furcifer petteri	ZSM 274/2004	male	-
Furcifer petteri	ZSM 315/2004	female	-
Furcifer petteri	ZSM 2136/2007	female	-
Furcifer petteri	ZSM 2137/2007	male	-
Furcifer petteri	ZSM 2141/2007	male	-
Furcifer petteri	ZSM 2142/2007	male	-
Furcifer petteri	ZSM 2160/2007	female	-
Furcifer petteri	ZSM 2161/2007	male	-
Furcifer petteri	ZSM 2022/2008	male	-
Furcifer petteri	ZSM 1706/2012	male	-
Furcifer petteri	ZSM 521/2000	female	-
Furcifer petteri	ZSM 785/2000	female	-
Furcifer petteri	ZSM 789/2000	male	-
Furcifer petteri	ZSM 790/2000	female	-
Furcifer petteri	ZSM 767/2003	female	-
Furcifer petteri	ZSM 859/2003	male	-
Furcifer petteri	ZSM 727/2014	male	-
Furcifer polleni	ZSM 37/2010	male	+
Furcifer polleni	ZSM 666/2000	male	+
Furcifer polleni	ZSM 667/2000	female	+
Furcifer rhinoceratus	ZSM 329/2014	-	-
Furcifer rhinoceratus	ZSM 532/2001	male	-
Furcifer rhinoceratus	ZSM 533/2001	male	-
Furcifer rhinoceratus	ZSM 534/2001	female	-
Furcifer rhinoceratus	ZSM 535/2001	-	-
Furcifer rhinoceratus	ZSM 787/2001	-	-
Furcifer timoni	ZSM 255/2004	female	-
Furcifer timoni	ZSM 256/2004	female	-
Furcifer timoni	ZSM 2103/2007	male	+
Furcifer timoni	ZSM 2023/2008	female	-
Furcifer verrucosus	ZSM 1063/2003	female	-
Furcifer verrucosus	ZSM 1064/2003	male	-
Furcifer verrucosus	ZSM 1065/2003	male	-
Furcifer verrucosus	ZSM 1066/2003	male	-

	Furcifer verrucosus	ZSM 1067/2003	male	-
	Furcifer verrucosus	ZSM 40/2004	female	-
	Furcifer verrucosus	ZSM 60/2004	male	-
	Furcifer verrucosus	ZSM 61/2004	male	-
	Furcifer verrucosus	ZSM 66/2009	male	-
	Furcifer verrucosus	ZSM 67/2009	female	-
	Furcifer verrucosus	ZSM 328/2014	male	-
	Furcifer verrucosus	ZSM 623/2000	male	-
	Furcifer verrucosus	ZSM 779/2000	-	-
	Furcifer verrucosus	ZSM 959/2003	male	-
	Furcifer viridis	ZSM 540/2001	-	-
	Furcifer viridis	ZSM 79/2006	male	-
	Furcifer viridis	ZSM 1729/2010	female	-
	Furcifer viridis	ZSM 1838/2010	male	-
	Furcifer viridis	ZSM 525/2014	female	-
	Furcifer viridis	ZSM 526/2014	male	-
	Furcifer viridis	ZSM 44/2016	male	-
	Furcifer willsii	ZSM 193/2004	male	-
	Furcifer willsii	ZSM 446/2005	male	-
	Furcifer willsii	ZSM 463/2010	female	-
	Furcifer willsii	ZSM 835/2014	male	-
	Furcifer willsii	ZSM 45/2016	female	-
Palleon				+
	Palleon lolontany	ZSM 1679/2012	female	+
Kinyongia				
	Kinyongia excubitor	ZSM 893/2000	male	+
	Kinyongia matschiei	ZSM 4746/2012	-	-
	Kinyongia multituberculata	ZSM 1/1920/1	male	+
	Kinyongia tavetana	ZSM 4747/2012	-	+
	Kinyongia tenue	ZSM 22/1915	-	+
	Kinyongia vosseleri	ZSM 2871/2007	male	+
	Kinyongia xenorhina	ZSM 4751/2012	male	+
Nadzikambia	no specimens available			
Rhampholeon				-
	Rhampholeon boulengeri	ZSM 4373/2012	male	-
	Rhampholeon platyceps	ZSM 16/1932/1	-	-
	Rhampholeon spectrum	ZSM 858/2000	female	-
Rieppeleon				-
	Rieppeleon brevicaudatus	ZSM 859/2000	-	-
	Rieppeleon kerstenii	ZSM 826/2000	-	-
Trioceros				
	Trioceros cristatus	ZSM 808/2000	male	-
	Trioceros deremensis	ZSM 671/1911	male	+
	Trioceros ellioti	ZSM 911/2000	-	-
	Trioceros fuelleborni	ZSM 4706/2012	-	-
	Trioceros hoehnelii	ZSM 907/2000	-	+
	Trioceros ituriensis	ZSM 893/2014	-	-
	Trioceros jacksonii	ZSM 839/2000	female	+
	Trioceros melleri	ZSM 814/2000	male	-
	Trioceros montium	ZSM 827/2000	male	-
	Trioceros oweni	ZSM 4719/2012	male	-
	Trioceros quadricornis	ZSM 933/2014	-	-
	Trioceros sternfeldi	ZSM 1766/2006	-	-
	Trioceros wiedersheimi	ZSM 855/2000	-	+

Table S7. Settings for micro-CT scans. Abbreviations: Diam, diamond target; Stand, standard (tungsten) target.

specimen	description	kV	mA	target	duration
C. amber ZSM 1041/2003	complete body	130	60	Diam	18 min
C. ambreense ZSM 1042/2003	complete body	130	50	Diam	18 min
C. andrigitraense ZSM 544/2001	complete body	130	60	Diam	18 min
C. andringitraense ZSM 554/2001	complete body	130	60	Diam	18 min
C. boettgeri ZSM 227/2002	chameleon head	130	80	Stand	30 min
C. boettgeri ZSM 272/2007	chameleon head	130	80	Stand	30 min
C. boettgeri ZSM 273/2007	chameleon head	130	80	Stand	30 min
C. boettgeri ZSM 36/1913	complete body	130	60	Diam	18 min
C. boettgeri ZSM 440/2000	chameleon head	130	80	Stand	30 min
C. boettgeri ZSM 441/2000	chameleon head	130	80	Stand	30 min
C. boettgeri ZSM 444/2000	chameleon head	130	80	Stand	30 min
C. boettgeri ZSM 551/2001	chameleon head	130	80	Stand	30 min
C. brevicorne ZSM 549/2001	complete body	130	50	Diam	18 min
C. cf. *fallax* ZSM 693/2009	chameleon head	130	80	Stand	30 min
C. cf. *gallus* ZSM 691/2009	chameleon head	130	80	Stand	30 min
C. cf. *linotum* ZSM 143/2016	chameleon head	130	80	Diam	30 min
C. cf. *vencesi* ZSM 620/2009	complete body	130	50	Diam	18 min
C. cf.*andringitraense* ZSM 0129/2005	complete body	130	60	Diam	18 min
C. cfmarojenze ZSM 462/2010	complete body	130	50	Diam	18 min
C. crypticum ZSM 455/2010	complete body	120	70	Diam	18 min
C. cuculatum ZSM 655/2014	complete body	130	50	Diam	18 min
C. fallax MNHN 1899.317	chameleon head	131	80	Diam	30 min
C. fallax ZSM 268/2010	complete body	130	60	Diam	18 min
C. fallax ZSM 685/2003	complete body	130	60	Diam	18 min
C. furcifer ZSM 225/2002	complete body	130	60	Diam	18 min
C. gallus ZSM 321/2000	complete body	130	60	Diam	18 min
C. gastrotaenia ZSM 226/2002	complete body	130	60	Diam	18 min
C. gehringi ZSM 2851/2010	complete body	130	80	Stand	30 min
C. glawi ZSM 2042/2008	complete body	131	60	Diam	18 min
C. globifer ZSM 141/2016	chameleon head	130	80	Diam	30 min
C. globifer ZSM 221/2002	complete body	130	50	Diam	18 min
C. globifer ZSM 221/2002	chameleon head	130	60	Diam	18 min
C. guibei 2842/2010	chameleon head	130	80	Stand	30 min
C. guibei ht SMF 50.354	chameleon head	130	80	Stand	30 min
C. guibei ZSM 1686/2012	chameleon head	130	80	Stand	30 min
C. guibei ZSM 1688/2012	chameleon head	130	80	Stand	30 min
C. guibei ZSM 2840/2010	chameleon head	130	80	Stand	30 min
C. guibei ZSM 2841/2010n	chameleon head	130	80	Stand	30 min
C. guibei ZSM 2849/2010	chameleon head	130	80	Stand	30 min
C. guibei ZSM 2855/2010	chameleon head	130	80	Stand	30 min
C. guillaumeti ZSM 440/2005	complete body	130	50	Diam	18 min
C. hafahafa DRV 06282	complete body	130	50	Diam	18 min
C. hafahafa ZSM 617/2009	complete body	130	50	Diam	18 min

C. hilleniusi ZSM 380/200	complete body	130	60	Diam	12 min
C. linotum ZSM 21/1923	complete body	120	80	Stand	30 min
C. malthe ZSM 71/2005	complete body	130	50	Diam	18 min
C. nasutum ZSM 134/2005	chameleon head	130	80	Stand	30 min
C. nasutum ZSM 135/2005	chameleon head	130	80	Stand	30 min
C. nasutum ZSM 35/2016	chameleon head	130	80	Diam	30 min
C. nasutum ZSM 441/2005	chameleon head	130	80	Stand	30 min
C. nasutum ZSM 443/2005	complete body	130	50	Diam	18 min
C. nasutum ZSM 553/2009	chameleon head	130	80	Stand	30 min
C. nasutum ZSM 618/2009	chameleon head	130	80	Stand	30 min
C. nasutum ZSM 619/2009	chameleon head	130	80	Stand	30 min
C. nasutum ZSM 735/2003	chameleon head	130	80	Stand	30 min
C. nasutum ZSM 924/2003	chameleon head	130	80	Stand	30 min
C. oshaughnessyi ZSM 1836/2010	complete body	130	50	Diam	18 min
C. parsoni ZSM 47/1990	complete body	130	60	Diam	36 min
C. peltierorum ZSM 1725/2010	complete body	130	50	Diam	18 min
C. peyrieasi ZSM 1727/2010	complete body	130	50	Diam	18 min
C. radamanus SMF 26394	complete body	130	80	Stand	30 min
C. tarzan ZSM 219/2010	complete body	120	80	Diam	18 min
C. tsaratanaense ZSM 1728/2010	complete body	130	50	Diam	18 min
C. vatosoa MRSN R1628	chameleon head	130	80	Stand	30 min
C. vatosoa SMF 26357	chameleon head	130	80	Stand	30 min
C. vencesi ZSM 50/2011	complete body	120	80	Diam	21 min
C. vencisi ZSM 50/2011	complete body	130	50	Diam	51 min
C. vohibola ZSM 645 2009	chameleon head	130	80	Stand	30 min
C. vohibola ZSM 645/2009	complete body	130	60	Diam	18 min
C. capuroni ZSM 5104/2005	complete body	130	60	Diam	18 min

Table S8. Equipment and settings of microscopy photography

Figs 2A,B	Volume renderings based on light micrographs (see corresponding section in material and methods): original 8 bit grayscale, jpg, 1390 x 1065 px @ 1.28 µm/px.<br>Processed with Adobe® Photoshop® CS6: edge length downsampled to 96% → 1051 x 1045 px @ 1.33 µm/px, converted to TIFF, 300 dpi.
Figs 2C,D	Light micrographs (10x): original 24 bit RGB, jpg, 2560 x 1920 px @ 342 nm/px. Processed with Adobe® Photoshop® CS6: edge length downsampled to 41%, height cropped from 788 to 710 px → 1051 x 710 px @ 834 nm/px, slight color correction (reduction of magenta), unsharp masked (radius 4px, strength 80%), converted to TIFF, 300 dpi.
Figs 2E,F	Light micrographs (40x): original 24 bit RGB, jpg, 1920 x 2560 px @ 85.2 nm/px.<br>Processed with Adobe® Photoshop® CS6: edge length downsampled to 54.7%, height cropped from 1400 to 710 px → 1051 x 710 px @ 156 nm/px, slight color correction (reduction of magenta), unsharp masked (radius 4px, strength 80%), converted to TIFF, 300 dpi.
Supplementary Fig. S1A	Light micrographs (10x): original 24 bit RGB, jpg, 2560 x 1920 px @ 342 nm/px.<br>Processed with Adobe® Photoshop® CS6: edge length downsampled to 41%, height cropped from 788 to 728 px → 1051 x 728 px @ 834 nm/px, slight color correction (reduction of magenta), unsharp masked (radius 4px, strength 80%), converted to TIFF, 300 dpi.
Supplementary Fig. S1B	Light micrographs (60x): original 24 bit RGB, jpg, 2560 x 1920 px @ 56.6 nm/px.<br>Processed with Adobe® Photoshop® CS6: edge length downsampled to 41%, height cropped from 788 to 728 px → 1051 x 728 px @ 138 nm/px, slight color correction (reduction of magenta), unsharp masked (radius 4px, strength 80%), converted to TIFF, 300 dpi.
Supplementary Figs S1C–E	TEM photographs (4400x): original 1376 x 1032 @ 15.8 nm/px, 8 bit grayscale, TIFF. Processed with Adobe® Photoshop® CS6: region of interest 515 x 942 px cropped (after rotation if necessary), upscaled to 692 x 1266 px (i.e. 134.369%) to match print area, final resolution 11.76 nm/px, brightness and contrast adjusted, 300 dpi.
